# Influence of Longitudinal Center of Mass Position on Load Distribution in High-Speed Quadrupedal Locomotion

**DOI:** 10.3390/biomimetics11080565

**Published:** 2026-08-07

**Authors:** Kaixin Lan, Lei Jiang, Yucheng Tao, Chaojie Fu, Yongbin Jin, Hongtao Wang

**Affiliations:** 1 Center for X-Mechanics, Zhejiang University, Hangzhou 310012, China; 2 State Key Laboratory of Fluid Power and Mechatronic System, Zhejiang University, Hangzhou 310012, China; 3 ZJU-Hangzhou Global Scientific and Technological Innovation Center, Zhejiang University, Hangzhou 311200, China

**Keywords:** quadruped robots, longitudinal center of mass, bio-inspired design, high-speed locomotion, dynamic load distribution

## Abstract

Existing quadruped robots typically place their center of mass (CoM) near the geometric center of the body to achieve structural symmetry and simplify control design. In contrast, many quadrupedal animals capable of agile running exhibit a pronounced anteriorly biased mass distribution, with the CoM located closer to the front of the body. This biological characteristic motivates a re-examination of whether a geometrically centered CoM necessarily corresponds to dynamically balanced loading between the fore- and hindlimbs during high-speed locomotion. To address this question, this study investigates the influence of longitudinal CoM position on load distribution during high-speed straight-line locomotion of quadruped robots. A unified analytical framework is established by combining whole-body force and pitch moment equilibrium, sagittal-plane kinematics, and Jacobian-based force-to-torque mapping, thereby linking longitudinal CoM position, foot-end support forces, and joint loads. Simulation validation is conducted on the Black Panther 2 quadruped robot using four central-body CoM configurations, denoted as ×0, ×5, ×10, and ×15. In the primary evaluation at 5 m/s, shifting the CoM forward from ×0 to ×15 reduces the absolute median fore–hindlimb differences in support force and joint torque by approximately 86.6% and 93.4%, respectively, indicating a transition from hindlimb-dominated loading toward cooperative load sharing between the fore and hindlimbs. Independent training runs with multiple random seeds further confirm the robustness of this load-redistribution trend to reinforcement learning variability. Consistent behavior is also observed at 8 m/s, while no evident degradation in turning response or locomotion stability is found under the tested turning and randomly generated rough-terrain conditions. These results demonstrate that a moderate forward shift of the longitudinal CoM can alleviate hindlimb load concentration and promote a more balanced fore–hindlimb load distribution, providing a theoretical basis for the morphological design and control optimization of high-speed quadruped robots.

## 1. Introduction

### 1.1. Research Background

Advances in high-torque actuation, massively parallel simulation, and learning-based control have enabled quadruped robots to perform high-speed running, robust traversal of complex terrain, and agile obstacle negotiation [1,2,3,4,5,6,7,8,9,10,11,12,13,14,15,16,17,18,19]. Despite the substantial progress in locomotion control, many quadruped robot models and mechanical layouts still employ approximately symmetric trunk structures and, for simplified modeling and mechanical design, place or approximate the center of mass (CoM) near the geometric middle of the trunk to reduce the complexity of structural analysis and controller development [20,21,22].

The morphological organization of natural quadrupeds provides intuitive inspiration for re-examining this conventional configuration. Biomechanical studies provide species-specific evidence that longitudinal CoM location and fore–hindlimb mechanics are not necessarily symmetric. In horses, the CoM is located anteriorly within the trunk, and its fore–aft location influences sagittal-plane pitching mechanics during locomotion [23,24]. In dogs, the forelimbs support approximately 60% of body mass, and experimental changes in fore–aft mass distribution alter fore–hindlimb force patterns during trotting [25,26,27]. In cats, the whole-body center of gravity is located closer to the forelimbs, consistent with a forelimb-biased weight distribution [28,29]. Collectively, these species-specific observations indicate that longitudinal mass distribution is a meaningful morphological factor in quadrupedal locomotion.

Against this biological background, the geometrically centered CoM commonly adopted in quadruped robots warrants further examination: does geometric symmetry necessarily lead to balanced dynamic loading between the fore- and hindlimbs during high-speed locomotion?

### 1.2. Recent Related Work

Learning-based control has recently become an important route for improving quadruped locomotion. Massively parallel deep reinforcement learning has markedly reduced the time required for policy training [6,7], while rapid motor adaptation enables a trained policy to respond online to previously unseen dynamics [9]. Perceptive policies and implicit terrain representations have further improved locomotion over rough or visually complex terrain [8,11]. For high-speed locomotion, Jin et al. proposed imitation–relaxation reinforcement learning to optimize competing objectives in stages and demonstrated stable running at 5.0 m/s on a Mini-Cheetah-like platform [19], whereas Margolis et al. learned rapid locomotion policies for demanding velocity commands [12]. Choi et al. further extended learned locomotion to deformable terrain by training a robot to infer and respond to terrain properties through proprioceptive interaction [17]. These studies primarily improve controller adaptation to the robot’s own state and the external environment while keeping the robot morphology fixed.

Recent work has further extended reinforcement learning from basic velocity tracking to agile skills and multi-gait locomotion. ANYmal Parkour learned to combine walking, climbing, crouching, and jumping skills for complex obstacle traversal [5]. Han et al. combined animal motion data, reinforcement learning, and generative pre-trained models to construct reusable motion knowledge for animal-like locomotion and downstream tasks [13]. Shafiee et al. combined deep reinforcement learning with a central pattern generator representation and observed the emergence of walk–trot and trot–pronk transitions under viability-related objectives [14]. Humphreys and Zhou subsequently incorporated gait-transition strategies, gait memory, and real-time motion adjustment into a deep reinforcement learning framework, enabling the robot to switch among multiple gaits and recover from instability [15]. For high-speed navigation, Kim et al. combined efficient foothold planning with a learned tracker to achieve dynamic traversal of complex, discrete terrain [18]. Together, these studies demonstrate that reinforcement learning can improve robustness, adaptability, gait diversity, and agile task execution in quadruped locomotion.

Recent studies have also considered physical design and control jointly, including the optimization of quadruped kinematic and actuator parameters [30], the co-design of parallel-elastic knee joints and their controllers [31], and the joint optimization of morphology and gait for small-scale legged robots [32]. These studies demonstrate the coupling between robot embodiment and control; however, the investigated variables remain concentrated mainly on the legs, elastic elements, and actuators rather than the longitudinal mass position of the whole robot.

Related research has also extended AI to the system-interface level. Drăgoi et al. combined camera-based offline gesture recognition with locally executed AI models. Although their platform was not developed for quadruped locomotion control, its modular on-device architecture provides a useful example of connecting local perception to robot command interfaces without continuous reliance on the cloud [33]. Overall, recent research continues to expand the locomotion controller itself, whereas the longitudinal mass distribution of the robot body is typically retained as a fixed design condition.

### 1.3. Research Gap

Despite these important advances, most intelligent locomotion studies treat robot morphology and inertial distribution as fixed conditions when improving controllers, perception modules, or gait strategies; correspondingly, recent morphology and control co-design studies have focused primarily on leg geometry, compliance, or actuator parameters. The longitudinal position of the whole-body CoM has received relatively limited systematic attention, particularly under high-speed forward locomotion.

### 1.4. Problem Statement

**Figure 1:**
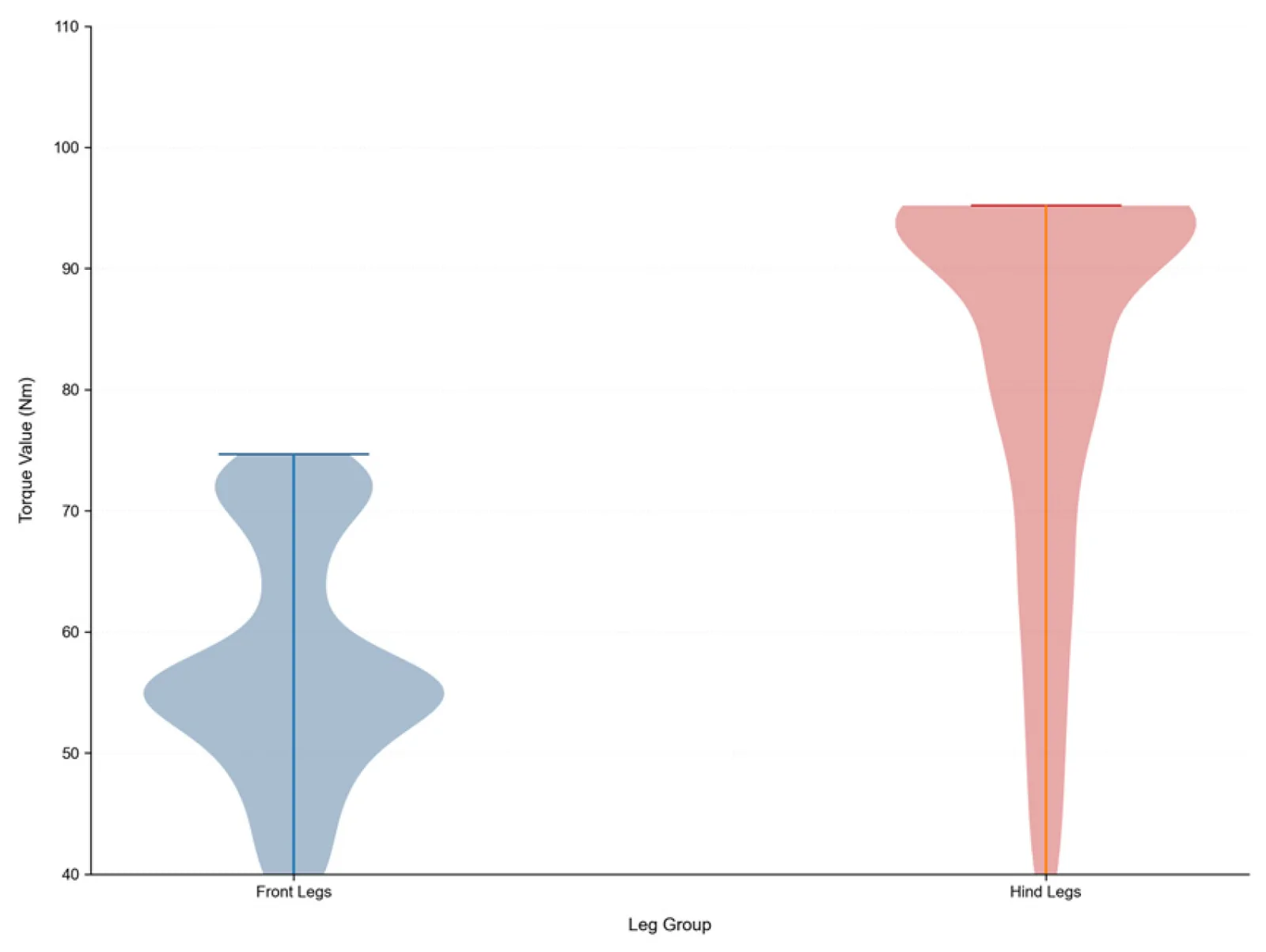
Fore–hindlimb calf joint torque distributions.

Preliminary simulations of the Black Panther 2 quadruped robot under a constant forward-velocity command of 5 m/s show that, when the CoM is located at the geometric center of the trunk, the torque demand of the hindlimb calf joints is markedly higher than that of the corresponding forelimb joints (Figure 1). This result indicates that a geometrically centered configuration may still exhibit a pronounced fore–hind load disparity during high-speed locomotion.

Based on this observation, the present study addresses the following questions: In high-speed straight-line locomotion, does locating the CoM at the geometric middle of the trunk necessarily imply dynamically balanced loading between the fore- and hindlimbs? What relationship exists between the longitudinal CoM position and the fore–hindlimb mechanical states during high-speed locomotion? Can a moderate forward shift of the CoM alleviate hindlimb load concentration and promote a more balanced fore–hind load distribution?

### 1.5. Research Objectives

This study aims to evaluate whether locating the CoM at the geometric middle of the trunk yields reasonably balanced mechanical loading between the fore- and hindlimbs during high-speed straight-line locomotion, and to quantify the influence of a moderate forward CoM shift on the redistribution of fore–hind support forces and joint torques. Under consistent simulation, training, and evaluation conditions, four longitudinal CoM configurations—×0, ×5, ×10, and ×15—are compared at 5 and 8 m/s.

### 1.6. Novelty of the Proposed Method

The novelty of this study lies in treating the longitudinal CoM position as an independent morphological variable under consistent training and evaluation conditions, and in analyzing its effects along the causal chain from whole-body pitch moment arms to fore–hind support force distribution and joint torque demand. A concise whole-body equilibrium-to-joint mapping is used to interpret the load transfer mechanism, while comparisons from ×0 to ×15 at 5 and 8 m/s reveal that a geometrically centered configuration may produce a fore–hind load disparity and show how a forward CoM shift moves the load and torque distributions toward a more balanced state. This morphology–load-focused comparison complements locomotion studies that primarily optimize controllers on a fixed robot body.

### 1.7. Main Contributions

1. Quantitatively re-examining the relationship between geometric symmetry and dynamic fore–hind load symmetry in high-speed quadrupedal locomotion, thereby treating a centered CoM as a design assumption that requires evaluation.
2. Using concise sagittal-plane equilibrium relations and foot-end Jacobian analysis to explain how the longitudinal CoM position changes pitch moment arms, fore–hind support force distribution, and joint torque demand.
3. Comparing the ×0, ×5, ×10, and ×15 configurations under controlled simulation conditions at 5 and 8 m/s. The resulting support-force and joint-torque distributions provide preliminary evidence of load asymmetry in the centered-CoM configuration and the load-balancing trend induced by a forward CoM shift.

## 2. Materials and Methods

### 2.1. Influence of Longitudinal CoM Position on Straight-Line Locomotion

#### 2.1.1. Whole-Body Equilibrium Analysis During Straight-Line Locomotion

**Figure 2:**
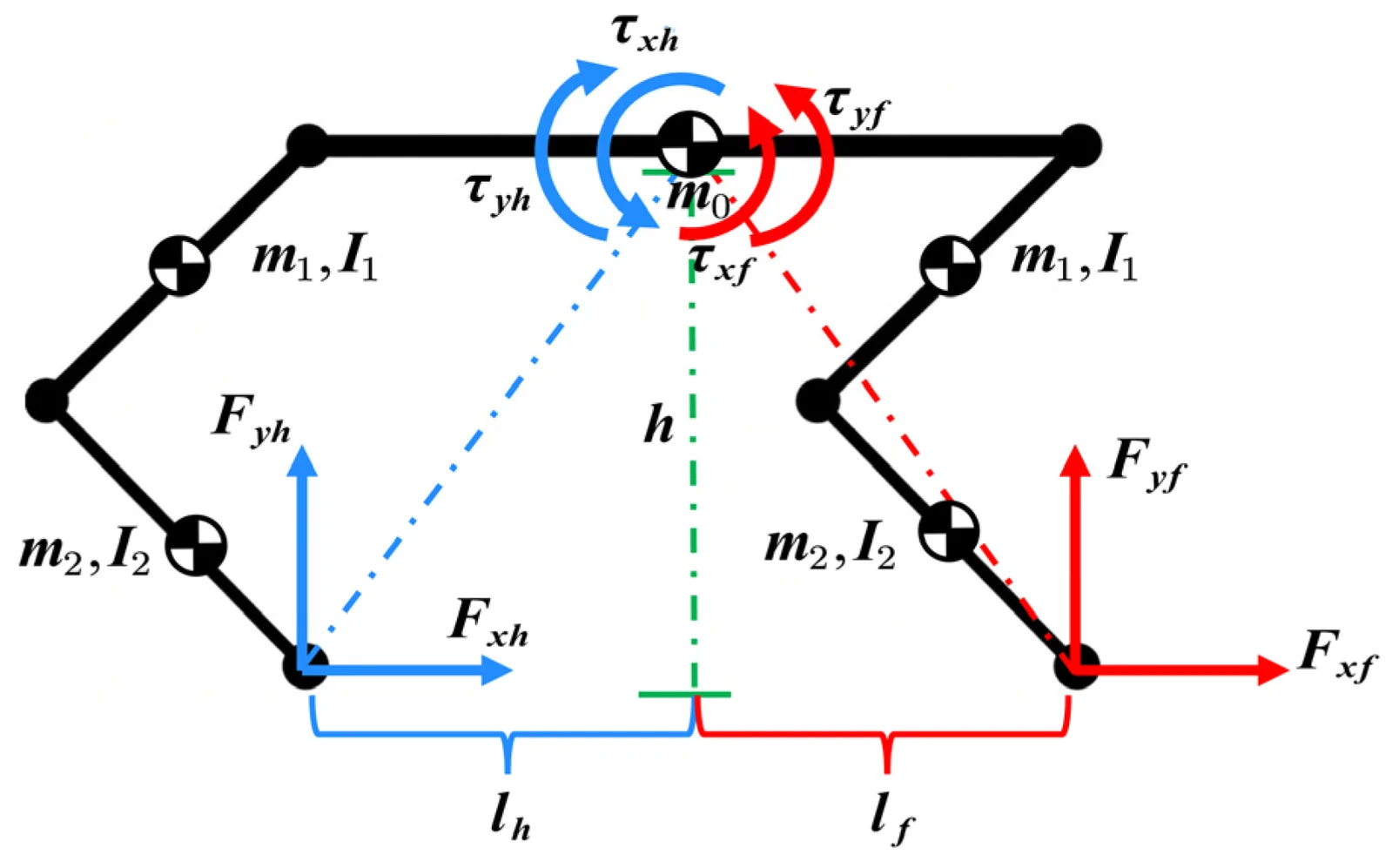
Simplified whole-body dynamics model for analyzing the influence of longitudinal CoM position.

During the straight-line locomotion of a quadruped robot, changes in the longitudinal position of the CoM alter the moment arm distribution between the body and the front and rear foot tips, thereby affecting the whole-body moment balance about the CoM (Figure 2). Since the ground reaction forces at the feet must simultaneously satisfy vertical force equilibrium and pitch moment equilibrium, their distribution between the front and rear limbs changes as the CoM position varies [34,35,36].

To characterize this mechanism, a simplified whole-body equilibrium model for an equivalent sagittal-plane support state is established. Let the total mass of the robot be *m*, the gravitational acceleration be *g*, the height of the CoM above the ground be *h*, and the horizontal distances from the hind and front foot tips to the CoM be $l_{h}$ and $l_{f}$, respectively. The forward locomotion direction is defined as the positive *x*-axis, and the vertically upward direction as the positive *y*-axis. The horizontal and vertical components of the ground reaction force acting on the hindlimbs are denoted by $F_{x}h$ and $F_{y}h$, whereas those acting on the front limbs are denoted by $F_{x}f$ and $F_{y}f$, respectively. 

The vertical force equilibrium of the robot is expressed as

(1) $$F_{yh}+F_{yf}=mg$$

Because the points of application of the horizontal ground reaction forces are located below the CoM, these force components generate an additional pitching moment about the CoM. According to the force and moment directions defined in Figure 2, the pitch moment equilibrium of the robot can be written as

(2) $$l_{f}F_{yf}+h(F_{xf}+F_{xh})=l_{h}F_{yh}$$

Accordingly, the coupled whole-body force and pitch moment equilibrium equations are

(3) $$\left\{\begin{array}{c} l_{f}F_{yf}+h(F_{xf}+F_{xh})=l_{h}F_{yh}\\ F_{yh}+F_{yf}=mg \end{array}\right.$$

Solving the above equations gives the analytical expressions for the vertical ground reaction forces of the hind and front limbs:

(4) $$\left\{\begin{array}{c} F_{yh}=\frac{mgl_{f}+h(F_{xf}+F_{xh})}{l_{h}+l_{f}}\\ F_{yf}=\frac{mgl_{h}-h(F_{xf}+F_{xh})}{l_{h}+l_{f}} \end{array}\right.$$

These analytical expressions show that the vertical ground reaction force distribution between the front and hindlimbs is jointly governed by the horizontal moment arms from the CoM to the two foot-contact locations, the CoM height, and the horizontal ground reaction forces. For the propulsion-dominated support state considered in this simplified analysis, the resultant horizontal ground reaction force acts in the direction of travel, namely:

(5) $$F_{xf}+F_{xh}>0$$

Because the line of action of this resultant force lies below the CoM, the induced pitching moment increases the vertical support force carried by the hindlimbs and reduces that carried by the front limbs. Therefore, when the CoM coincides with the geometric center of the body, such that $l_{h}=l_{f}$, the analytical expressions directly yield $F_{y}h>F_{y}f$.

Hence, even for a geometrically symmetric body with a centered CoM, the dynamic load distribution remains asymmetric during the forward locomotion state considered here, with the hindlimbs carrying the larger vertical support load. The geometric center therefore does not coincide with the dynamically balanced load-distribution position.

As the CoM is shifted forward along the longitudinal axis of the body, $l_{h}$ increases whereas $l_{f}$ decreases. Under otherwise comparable locomotion conditions, the analytical expressions consequently predict a reduction in $F_{y}h$ and an increase in $F_{y}f$. A moderate forward CoM shift therefore alleviates the concentration of load on the hindlimbs under the centered-CoM configuration and promotes a more balanced distribution of vertical support forces between the front and hindlimbs

#### 2.1.2. Sagittal-Plane Kinematic Modeling and Joint Dynamics Mapping

**Figure 3:**
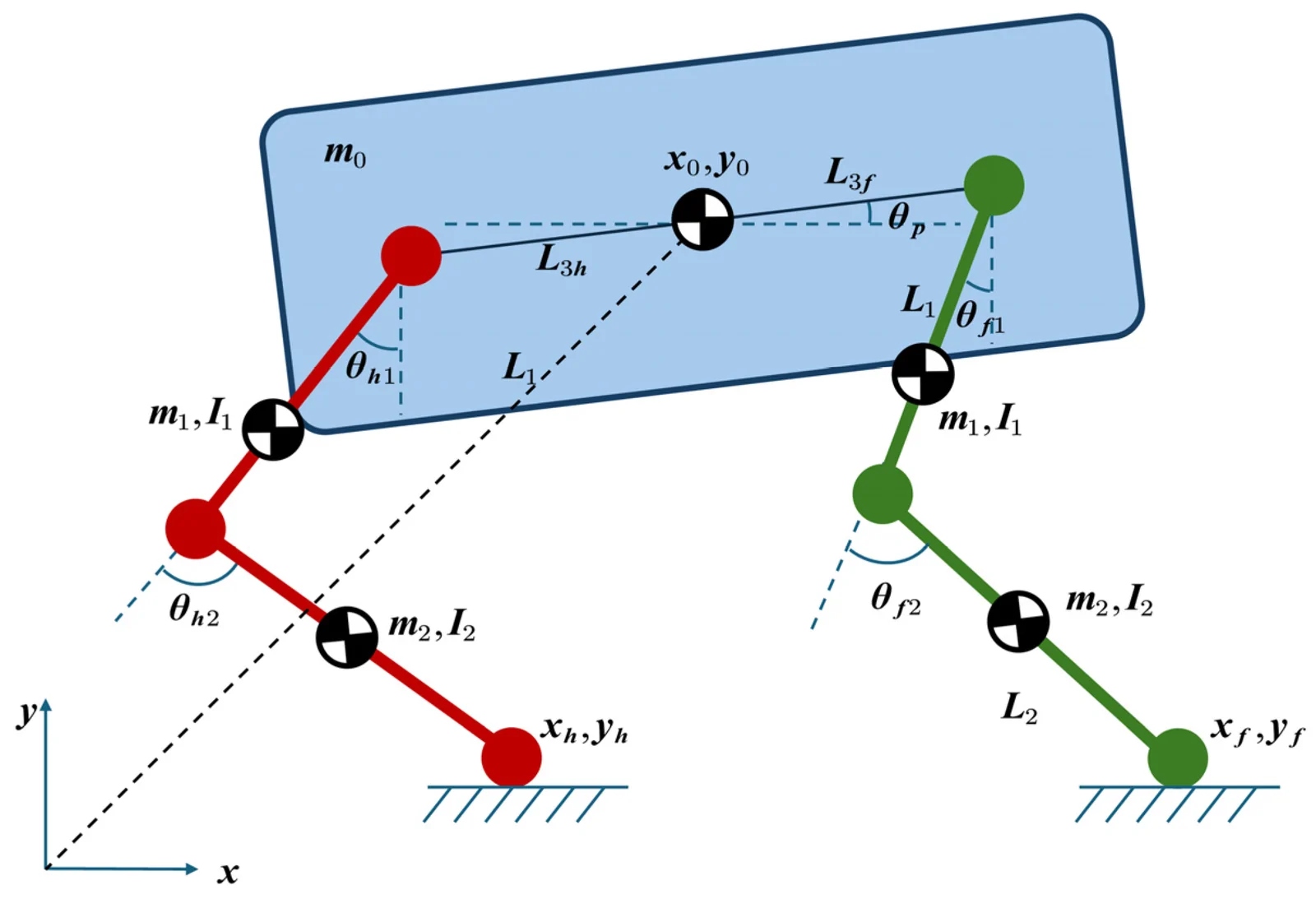
Sagittal-plane kinematic model of the quadruped robot for foot-end motion analysis and Jacobian-based force mapping. The trunk is shown in blue, while the hindlimb and forelimb kinematic chains are shown in red and green, respectively.

To construct the dynamic mapping relationship of the quadruped robot, a forward kinematics model of the body and a single leg is first established in the sagittal plane (Figure 3). The global reference coordinate system $\left\{W\right\}$ is defined, with the X-axis horizontal pointing to the right as positive and the Y-axis vertical pointing upward as positive. Let the Cartesian space coordinates of the robot’s center of mass (CoM) be $\left(x_{0}\;,\;y_{0}\right)$, and let the pitch angle of the body about the CoM be $\theta_{p}$ (with counterclockwise rotation defined as positive). The offset distances from the CoM to the rear and front hip joints are independently defined as $L_{3h}$ and $L_{3f}$, respectively. In addition, the physical lengths of the thigh and shank links are denoted by $L_{1}$ and $L_{2}$, respectively. The generalized coordinate variables of the system are defined as

(6) $$\begin{array}{c} q={\left[x_{0},y_{0},\theta_{p},\theta_{h1},\theta_{h2},\theta_{f1},\theta_{f2}\right]}^{T} \end{array}$$

where $\theta_{h1}$ and $\theta_{h2}$ represent the hip and knee joint angles of the rear leg, respectively, and $\theta_{f1}$ and $\theta_{f2}$ represent the corresponding joint angles of the front leg.

Based on the above geometric definitions and the spatial relationships among the links, the forward kinematic equations of the rear leg foot tip (h) can be expressed as

(7) $$\begin{array}{c} \left\{\begin{array}{c} x_{h}=x_{0}-L_{3h}{\cos\theta }_{p}-L_{1}{\sin\theta }_{h1}+L_{2}\sin\left(\theta_{h2}-\theta_{h1}\right)\\ y_{h}=y_{0}-L_{3h}{\sin\theta }_{p}-L_{1}{\cos\theta }_{h1}-L_{2}\cos\left(\theta_{h2}-\theta_{h1}\right) \end{array}\right. \end{array}$$

Similarly, the forward kinematic equations of the front leg foot tip are derived as

(8) $$\begin{array}{c} \left\{\begin{array}{c} x_{f}=x_{0}+L_{3f}{\cos\theta }_{p}-L_{1}{\sin\theta }_{f1}+L_{2}\sin\left(\theta_{f2}-\theta_{f1}\right)\\ y_{f}=y_{0}+L_{3f}{\sin\theta }_{p}-L_{1}{\cos\theta }_{f1}-L_{2}\cos\left(\theta_{f2}-\theta_{f1}\right) \end{array}\right. \end{array}$$

The above analytical expressions of the foot tip positions precisely map the robot’s joint configuration space to the Cartesian task space. To further investigate the mapping mechanism from ground reaction forces to the joint space, the system’s Jacobian matrix J(q) is introduced, which is defined as the linear mapping between the foot tip velocity and the generalized velocity [37,38,39,40]. By taking the partial derivatives of the forward kinematic equations of the rear leg foot tip (h), the local Jacobian matrix $J_{h}$ corresponding to the rear leg foot tip can be obtained as

(9) $$J_{h}=\left[\begin{array}{ccccccc} 1 & 0 & L_{3h}{\sin\theta }_{p} & -L_{1}{\cos\theta }_{h1}-L_{2}\cos\left(\theta_{h2}-\theta_{h1}\right) & L_{2}\cos\left(\theta_{h2}-\theta_{h1}\right) & 0 & 0\\ 0 & 1 & -L_{3h}{\cos\theta }_{p} & L_{1}{\sin\theta }_{h1}-L_{2}\sin\left(\theta_{h2}-\theta_{h1}\right) & L_{2}\sin\left(\theta_{h2}-\theta_{h1}\right) & 0 & 0 \end{array}\right]$$

Similarly, the local Jacobian matrix $J_{f}$ corresponding to the front leg is derived as

(10) $$J_{f}=\left[\begin{array}{ccccccc} 1 & 0 & -L_{3f}{\sin\theta }_{p} & 0 & 0 & -L_{1}{\cos\theta }_{f1}-L_{2}\cos\left(\theta_{f2}-\theta_{f1}\right) & L_{2}\cos\left(\theta_{f2}-\theta_{f1}\right)\\ 0 & 1 & L_{3f}{\cos\theta }_{p} & 0 & 0 & L_{1}{\sin\theta }_{f1}-L_{2}\sin\left(\theta_{f2}-\theta_{f1}\right) & L_{2}\sin\left(\theta_{f2}-\theta_{f1}\right) \end{array}\right]$$

By the principle of virtual work and assuming no external torque disturbances, the foot force $F_{h}=[F_{xh},F_{yh}{]}^{T}$ maps to the joint torques $\tau_{h}$ via $\tau_{h}=J_{h}^{T}F_{h}$. Extracting the transposed columns of $J_{h}$ associated with the rear leg joints ($\theta_{h1}$ and $\theta_{h2}$) yields the analytical expressions for the rear hip torque $\tau_{h,\mathrm{hip}}$ and knee torque $\tau_{h,\mathrm{knee}}$:

(11) $$\left\{\begin{array}{l} \tau_{h,hip}=\left[-L_{1}{\cos\theta }_{h1}-L_{2}\cos\left(\theta_{h2}-\theta_{h1}\right)\right]F_{xh}+\left[L_{1}{\sin\theta }_{h1}-L_{2}\sin\left(\theta_{h2}-\theta_{h1}\right)\right]F_{yh}\\ \tau_{h,knee}=\left[L_{2}\cos\left(\theta_{h2}-\theta_{h1}\right)\right]F_{xh}+\left[L_{2}\sin\left(\theta_{h2}-\theta_{h1}\right)\right]F_{yh} \end{array}\right.$$

Under general locomotion conditions, the joint torques are jointly determined by the instantaneous horizontal and vertical ground reaction forces and the joint configuration. Therefore, without specifying the instantaneous foot–ground interaction forces, the analytical model alone cannot determine the relative magnitudes and distribution of the fore- and hindlimb joint torques throughout the complete locomotion cycle.

To further reveal how the support force imbalance is transmitted to the joint loads under high propulsion demands, a limiting propulsion condition is considered, in which the friction utilization at the foot approaches its upper bound. For the hindlimb, the horizontal ground reaction force is approximated by

(12) $$F_{xh}=\mu F_{yh}.$$

Substituting this into the rear leg torque expressions and factoring out $F_{yh}$ yields the rear limb joint torque distribution model:

(13) $$\begin{array}{c} \left\{\begin{array}{l} \tau_{h,hip}=\left\{-\mu \left[L_{1}{\cos\theta }_{h1}+L_{2}\cos\left(\theta_{h2}-\theta_{h1}\right)\right]+L_{1}{\sin\theta }_{h1}-L_{2}\sin\left(\theta_{h2}-\theta_{h1}\right)\right\}F_{yh}\\ \tau_{h,knee}=\left[\mu L_{2}\cos\left(\theta_{h2}-\theta_{h1}\right)+L_{2}\sin\left(\theta_{h2}-\theta_{h1}\right)\right]F_{yh} \end{array}\right. \end{array}$$

Similarly, based on the front leg kinematics and the Jacobian matrix $J_{f}$, the mapping between the front leg joint torques and $F_{yf}$ is obtained as

(14) $$\left\{\begin{array}{l} \tau_{f,hip}=\left\{-\mu \left[L_{1}{\cos\theta }_{f1}+L_{2}\cos\left(\theta_{f2}-\theta_{f1}\right)\right]+L_{1}{\sin\theta }_{f1}-L_{2}\sin\left(\theta_{f2}-\theta_{f1}\right)\right\}F_{yf}\\ \tau_{f,knee}=\left[\mu L_{2}\cos\left(\theta_{f2}-\theta_{f1}\right)+L_{2}\sin\left(\theta_{f2}-\theta_{f1}\right)\right]F_{yf} \end{array}\right.$$

These expressions describe how the vertical support forces are transmitted to the joint loads under a high-propulsion boundary condition. For a given joint configuration, the posture-dependent terms remain fixed, and the joint torques vary linearly with the corresponding vertical support forces. It should be emphasized that the relations $F_{xh}=\mu F_{yh}$ and $F_{xf}=\mu F_{yf}$ are introduced only to characterize the loading boundary near the friction limit and are not assumed to hold throughout the complete locomotion cycle. The actual joint torque distributions over the complete locomotion cycle are subsequently evaluated using the foot-end forces, joint configurations, and joint torques obtained from the dynamic simulations.

#### 2.1.3. Conclusion on the Influence of Longitudinal CoM Position on Joint Torques

Under the limiting propulsion condition described above, for a given joint configuration and friction coefficient, the posture-dependent terms in the joint torque expressions become fixed coefficients. Accordingly, the fore- and hindlimb joint torques can be written in the following unified form:

(15) $$\left\{\begin{array}{c} \tau_{h,j}=K_{h,j}(\theta_{h},\;\mu )F_{yh}\\ \tau_{f,j}=K_{f,j}(\theta_{f},\;\mu )F_{yf} \end{array}\right.$$

where *j* ∈ {hip, knee}, and $K_{h,j}$ and $K_{f,j}$ denote the force-to-torque mapping coefficients determined by the leg geometry, joint configuration, and friction coefficient.

Combining these relations with the vertical support force expressions derived above yields

(16) $$\left\{\begin{array}{c} \tau_{h,j}=K_{h,j}(\theta_{h},\;\mu )\frac{mg(l_{f}+\mu h)}{l_{h}+l_{f}}\\ \tau_{f,j}=K_{f,j}(\theta_{f},\;\mu )\frac{mg(l_{h}-\mu h)}{l_{h}+l_{f}} \end{array}\right.$$

These expressions directly establish the relationship between the longitudinal CoM position and the joint loads. For a robot with identical fore- and hindlimb structures and comparable support configurations, the force-to-torque mapping coefficients are of similar magnitude; therefore, the difference in joint loads is primarily governed by the corresponding vertical support force distribution. When the CoM is located at the geometric center of the body, the higher hindlimb support force is transmitted through the Jacobian mapping into a higher joint torque demand on the hindlimb.

When the CoM is moderately shifted forward, $l_{h}\;$ increases while ${\;l}_{f}\;$ decreases. Consequently, the hindlimb support force and the corresponding joint torques decrease, whereas those of the forelimbs increase. Therefore, a forward shift of the longitudinal CoM redistributes the joint loads originally concentrated on the hindlimbs between the fore- and hindlimbs, thereby reducing the risk of premature torque saturation in the hindlimb motors.

### 2.2. Simulation Experiment

To verify the correctness of the previously established dynamic theoretical model of longitudinal CoM regulation and to quantitatively evaluate its influence on straight-line locomotion performance, this section takes the Black Panther 2 quadruped robot model as the common simulation platform and designs comparative simulation experiments under different longitudinal CoM configurations. By constructing a large-scale parallel reinforcement learning environment [6,7] and ensuring the robustness of the control policy to heterogeneous dynamic variants, cross-validation between the theoretical analysis and high-fidelity simulation data is achieved. The policy implementation further follows related robust policy-optimization and latent state-estimation strategies for legged locomotion [8,9,10,11,12,41,42].

#### 2.2.1. Network Architecture

**Figure 4:**
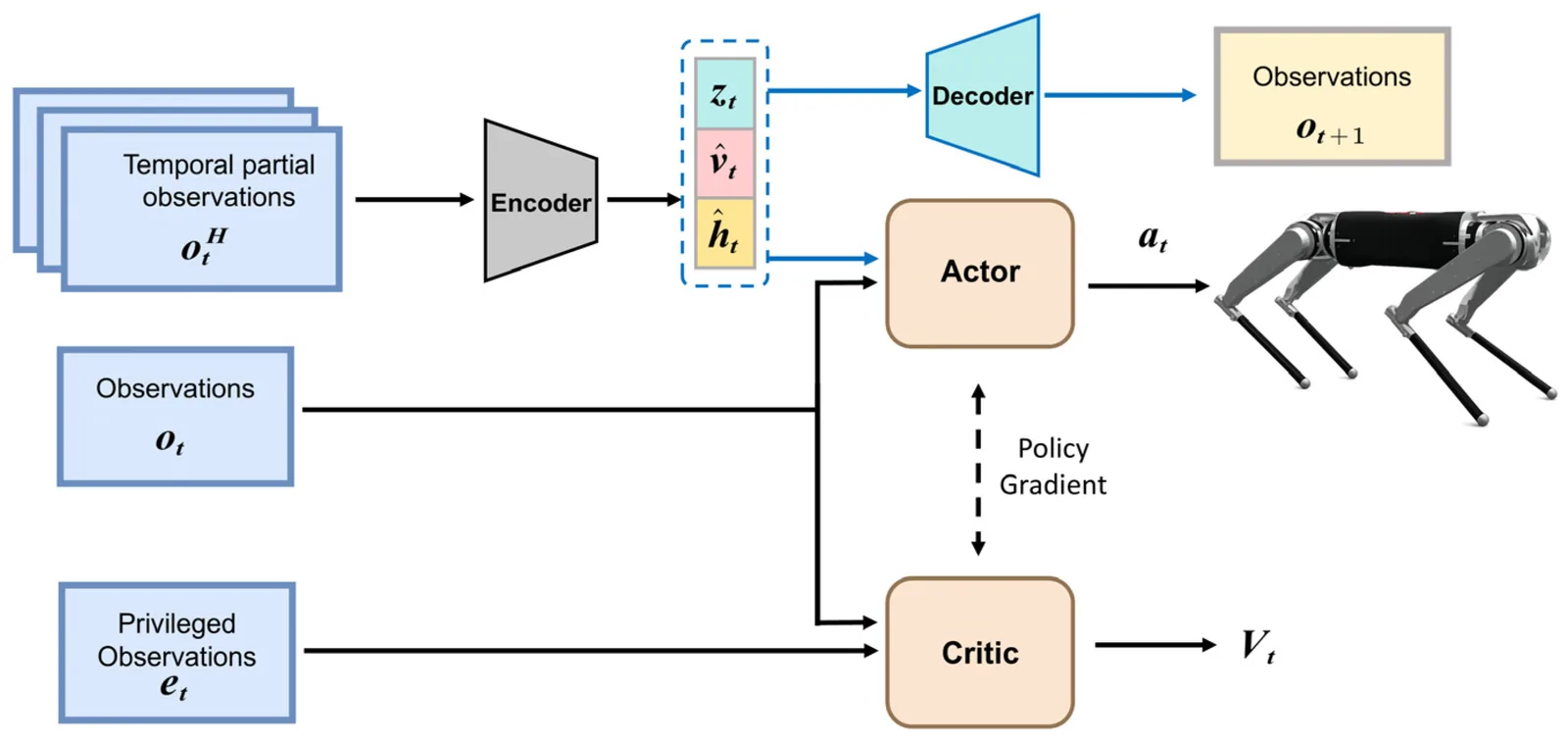
Asymmetric actor–critic framework with VAE-based state estimation for dynamic quadruped locomotion control.

To enable the robot to autonomously explore the physical limits of locomotion and achieve stable, high-speed, and heavy-load control over a wide range of commands, we employ an asymmetric actor–critic architecture as the algorithmic backbone [8,41], as shown in Figure 4.

At the beginning of training, the system first learns basic locomotion policies within a conventional command range. The initial command ranges for forward velocity, lateral velocity, and yaw angular velocity are all set to [−1, 1]. As the policy’s tracking performance improves, a reward-threshold-based grid curriculum progressively expands the sampling ranges of the forward velocity and yaw angular velocity commands through an adaptive curriculum mechanism [7,12], eventually reaching $[-2,\;10]\;m\;s^{-1}$ and $[-2,\;2]\;rad\;s^{-1}$, respectively. The lateral velocity command range remains fixed at $[-1,\;1]\;m\;s^{-1}$ throughout training. This curriculum mechanism enables the policy to gradually transition from basic locomotion to high-speed straight-line locomotion.

To meet the demand for rapid policy response in highly dynamic conditions, the system integrates a variational autoencoder (VAE) [42] as a state estimator to extract environmental and ego-state features from the historical observation data $o_{t}^{H}$. This estimator infers a 16-dimensional latent variable $z_{t}\in R^{16}$ in real time and explicitly estimates key physically meaningful states, including the body linear velocity $v_{t}$ and the base height $h_{t}$. To jointly optimize explicit state estimation and implicit environmental feature extraction, the network weights of this state estimator are updated via a hybrid loss function:

(17) $$L_{total}=L_{est}+L_{VAE}$$

where $L_{\mathrm{est}}$ is the supervised learning loss for the explicit states, defined as the mean squared error (MSE) between the predicted state values (${\hat{v}}_{t},{\hat{h}}_{t}$) and the ground-truth labels ($v_{t},h_{t}$) provided by the simulator

(18) $$L_{est}=\mathrm{MSE}\left({\hat{v}}_{t},v_{t}\right)+\mathrm{MSE}({\hat{h}}_{t},h_{t})$$

$\;L_{VAE}\;$ is the variational autoencoder loss, consisting of two components: the reconstruction loss for reconstructing the environmental dynamics at the next time step, and the distribution regularization term for constraining the latent variable feature space:

(19) $$L_{VAE}=\mathrm{MSE}\left({\tilde{o}}_{t+1},o_{t+1}\right)+\beta \;D_{KL}\left(q\left(z_{t}|o_{t}^{H}\right)\;\right\|\;p\left(z_{t}\right))$$

In the above formula, ${\tilde{o}}_{t+1}$ is the reconstructed value of the egocentric observation at the next time step, and β is a weighting coefficient that balances the reconstruction accuracy and the degree of latent space feature disentanglement; the prior distribution $p(z_{t})$ is typically set to a standard normal distribution.

The policy network and the value network adopt an asymmetric input design to fully exploit the privileged information available in the simulation environment [8,9,10,11,12,41]:

- **Policy network (actor):** The actor receives a 45-dimensional proprioceptive observation $o_{t}$, a 3-dimensional estimated body linear velocity ${\hat{v}}_{t}$, a scalar estimate of the base height ${\hat{h}}_{t}$, and a 16-dimensional latent variable $z_{t}$, resulting in a total input dimension of 65. The proprioceptive observation $o_{t}$ comprises the body angular velocity, the gravity vector projected into the body frame, velocity commands, joint positions, joint velocities, and the previous action. The actor is implemented as a multilayer perceptron with three hidden layers containing 512, 256, and 128 units, respectively, followed by a 12-dimensional action output layer. The exponential linear unit (ELU) activation function is applied to all hidden layers.
- **Value network (critic):** The critic receives the current 45-dimensional proprioceptive observation $o_{t}$, together with the 3-dimensional ground-truth body linear velocity $v_{t}$ and the scalar ground-truth base height $h_{t}$, which are available only during simulation training. The total input dimension is therefore 49. The critic is implemented as a multilayer perceptron with three hidden layers containing 512, 256, and 128 units, respectively, followed by a scalar value output layer. ELU activation functions are applied to all hidden layers.
- **VAE architecture:** The state estimator stacks five consecutive 45-dimensional proprioceptive observations and flattens them into a 225-dimensional historical observation vector $o_{t}^{H}$. A shared encoder with two hidden layers containing 128 and 64 units extracts a 64-dimensional history representation. This shared representation is then passed through four independent linear output heads with dimensions 64–16, 64–16, 64–3, and 64–1 to predict the mean and log-variance of the 16-dimensional latent distribution, the 3-dimensional body linear velocity, and the scalar base height, respectively. The 16-dimensional latent variable is sampled using the reparameterization trick and is provided to the actor together with the explicit state estimates. The decoder receives a 20-dimensional input formed by concatenating the latent variable, estimated body linear velocity, and estimated base height. It contains two hidden layers with 64 and 128 units, respectively, followed by a 45-dimensional reconstruction output layer. ELU activation functions are used in all hidden layers.

#### 2.2.2. Training Details

This section summarizes the training and physical-simulation settings used in the comparative experiments. The reward functions, PPO hyperparameters, learning rates, random seeds, contact parameters, robot inertial properties, motor limits, and CoM-shift implementation are specified to facilitate reproducibility. All four CoM configurations are trained and evaluated using the same algorithmic settings and physical parameters, with the longitudinal position of the central-body inertial origin being the only morphological parameter varied. The reward functions and their corresponding weights are summarized in Table 1.

**PPO parameters.** Training was performed using 4096 parallel environments, with 24 simulation steps collected from each environment per policy update. Each update comprised five learning epochs and four mini-batches. The PPO clipping ratio was set to 0.2, the discount factor to γ = 0.99, and the generalized advantage estimation parameter to λ = 0.95. The value-loss coefficient, entropy coefficient, and maximum gradient norm were set to 1.0, 0.01, and 1.0, respectively. The maximum number of policy updates was 20,000. The simulation time step was 0.005 s, and a control decimation factor of 4 was used, corresponding to a policy frequency of 50 Hz.

**Learning rates and random seeds.** The learning rates for the actor and critic were both set to 3 × 10^−4^, while the VAE learning rate was set to 1 × 10^−3^. The weighting coefficient of the Kullback–Leibler divergence term was set to β = 0.001. All experiments were implemented in Python 3.8.20 using NumPy 1.24.3 and PyTorch 2.4.1 with CUDA 12.4. For each CoM configuration, six independent training runs were conducted using random seeds {1, 2, 3, 4, 5, 6}, with each seed consistently applied across Python, NumPy, PyTorch, and CUDA.

**Contact parameters.** Training was conducted in NVIDIA Isaac Gym Preview 4 (version 1.0rc4) [6] with friction randomization enabled. The robot–ground friction coefficient was sampled from [0.3, 1.00], and the coefficient of restitution was set to 0. In MuJoCo 3.1.2, the two tangential sliding-friction coefficients of the foot–ground contact pair were set to 0.75 and 0.50, the torsional friction coefficient to 0.005, and both rolling-friction coefficients to 0.0001. The MuJoCo model did not directly specify a coefficient of restitution; instead, the nonelastic contact response was described using solref = [0.008, 1.50] and solimp = [0.5, 0.8, 0.01].

**Robot mass and inertia.** The total robot mass was 28.683 kg. The central body link (the URDF base link connecting the four legs) had a mass of 10.383 kg. Its inertia tensor, expressed about its own center of mass, was

I = [[0.111,0.00045,0.000048],[0.00045,0.661,0],[0.000048,0,0.695]] kg·m^2^

The mass and inertia tensor of every link were kept unchanged across the four CoM configurations.

**Motor limits.** A motor speed–torque envelope was enforced during training. The hip-abduction, hip-pitch, and knee joints used identical limits: a maximum torque of 95.2 N·m, a constant-torque cutoff speed of 195/8.5 = 22.94 rad·s^−1^, and a no-load speed of 278/8.5 = 32.71 rad·s^−1^. Above the cutoff speed, the available torque decreased linearly with joint speed and reached zero at the no-load speed.

**CoM-shift method.** The longitudinal mass-distribution adjustment was implemented by modifying the inertial origin of the central body link in the URDF. Specifically, the x-coordinate in <inertial><origin xyz=“x 0 0”> was set to 0, 0.05, 0.10, and 0.15 m, respectively, with the positive x-direction pointing forward. These four configurations are denoted as ×0, ×5, ×10, and ×15 throughout this paper. The labels therefore represent the longitudinal offsets applied to the central-body inertial origin, rather than identical numerical shifts of the full-system CoM. Except for this inertial-origin position, the total robot mass, link inertia tensors, geometric dimensions, and joint locations were unchanged, and the selected offset remained fixed throughout training.

#### 2.2.3. Experimental Setup

The control policies were trained on the NVIDIA Isaac Gym platform [6], where 4096 independent Black Panther 2 robot environments are instantiated in parallel, with a total training duration of approximately 6 h.

After training, the control policies corresponding to the different CoM configurations were transferred zero-shot to the MuJoCo environment for the primary high-speed straight-line locomotion evaluation under a constant forward-velocity command of 5 m/s. For each CoM configuration, 100 independent tests were conducted, each lasting 30 s. The relevant dynamic data during steady-state locomotion were statistically analyzed to quantitatively evaluate the influence of the longitudinal CoM position on fore–hindlimb load distribution. In addition, the same policy-transfer and evaluation procedures were applied at a forward velocity of 8 m/s. The fore–hindlimb support force and joint torque distributions were further compared to examine whether the observed load-redistribution trend remained valid at a higher locomotion speed.

To evaluate the influence of reinforcement learning variability on the results, six independent training runs were conducted for each of the ×0, ×5, ×10, and ×15 configurations using random seeds 1–6. Each policy was trained from random initialization using the same network architecture, training parameters, number of training iterations, and environment settings. After training, all policies were evaluated under the same straight-line locomotion condition. For each independent run, the median support force and median joint torque over the steady-state evaluation data were first calculated as representative statistics. The mean and standard deviation of these per-run median values were then computed across the six random seeds for each CoM configuration.

Supplementary turning and randomly generated rough-terrain evaluations were conducted in Isaac Gym [6]. The turning evaluation recorded the actual yaw-rate responses of the different CoM configurations over a range of forward velocities to compare the influence of the longitudinal CoM position on the speed–turning response. In the rough-terrain evaluation, the policies trained exclusively on flat terrain were directly deployed on randomly generated rough height fields without terrain-specific retraining. The root mean square values of the body pitch angle and vertical velocity were used to characterize the locomotion states of the different CoM configurations under rough-terrain conditions.

## 3. Results

### 3.1. Support Force Distribution

To validate the previously established theoretical mapping from the longitudinal CoM position to the foot-end support forces and joint torques, this section statistically analyzes the steady-state straight-line locomotion results obtained at 5 m/s. The four previously defined CoM configurations, ×0, ×5, ×10, and ×15, are first compared in terms of their fore–hindlimb normal support force distributions, while the corresponding joint torque, energy consumption, and extreme angular impulse are examined in the subsequent subsections. Since the foot-end support forces are transmitted primarily to the calf joint and contribute relatively little to the hip joint, the torque analysis focuses on the calf joint to more effectively characterize how the support forces influence the lower-limb actuation joints.

**Figure 5:**
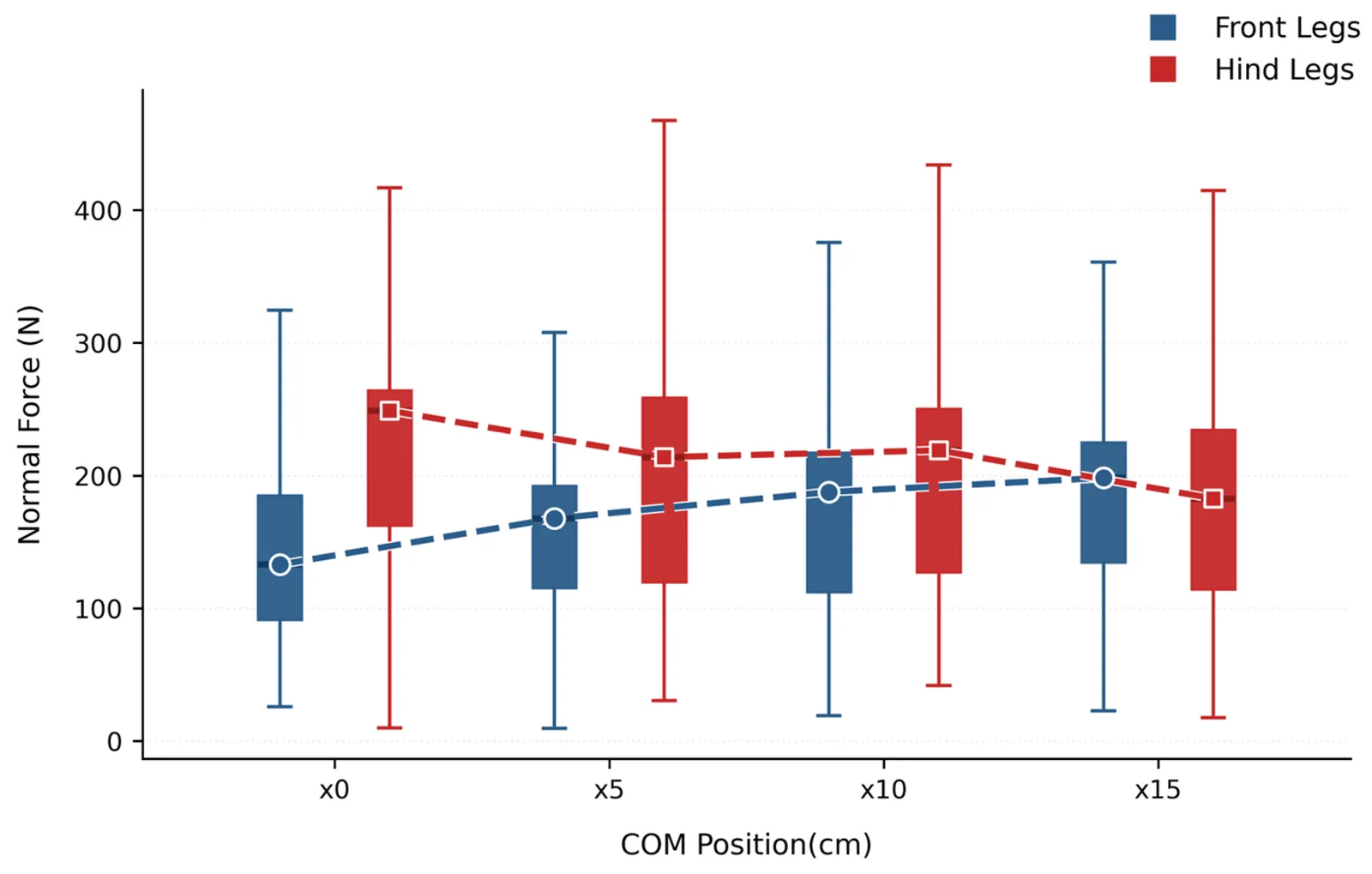
Comparison of normal support force distributions between the fore and hindlimbs under different CoM positions. The blue and red dashed lines connect the median values of the forelimb and hindlimb distributions, respectively.

As shown in Figure 5, when the CoM is at the initial position ×0, the normal support force of the rear limb is markedly higher than that of the front limb, indicating that during high-speed straight-line locomotion, the inertial load transfer causes the rear limb to bear a greater vertical support demand. As the CoM shifts forward from ×0 to ×15, the median level of the front limb normal support force gradually increases, while that of the rear limb decreases overall, and the gap between the two continuously narrows. This phenomenon directly validates the prediction of the whole-body mechanical equilibrium model established earlier: the forward shift of the CoM alters the horizontal moment arm distribution of the front and rear foot tips relative to the CoM, thereby compensating for the load transfer effect that concentrates toward the rear limb and driving the normal support forces of the front and rear limbs toward a more balanced distribution.

### 3.2. Joint Torque Distribution

**Figure 6:**
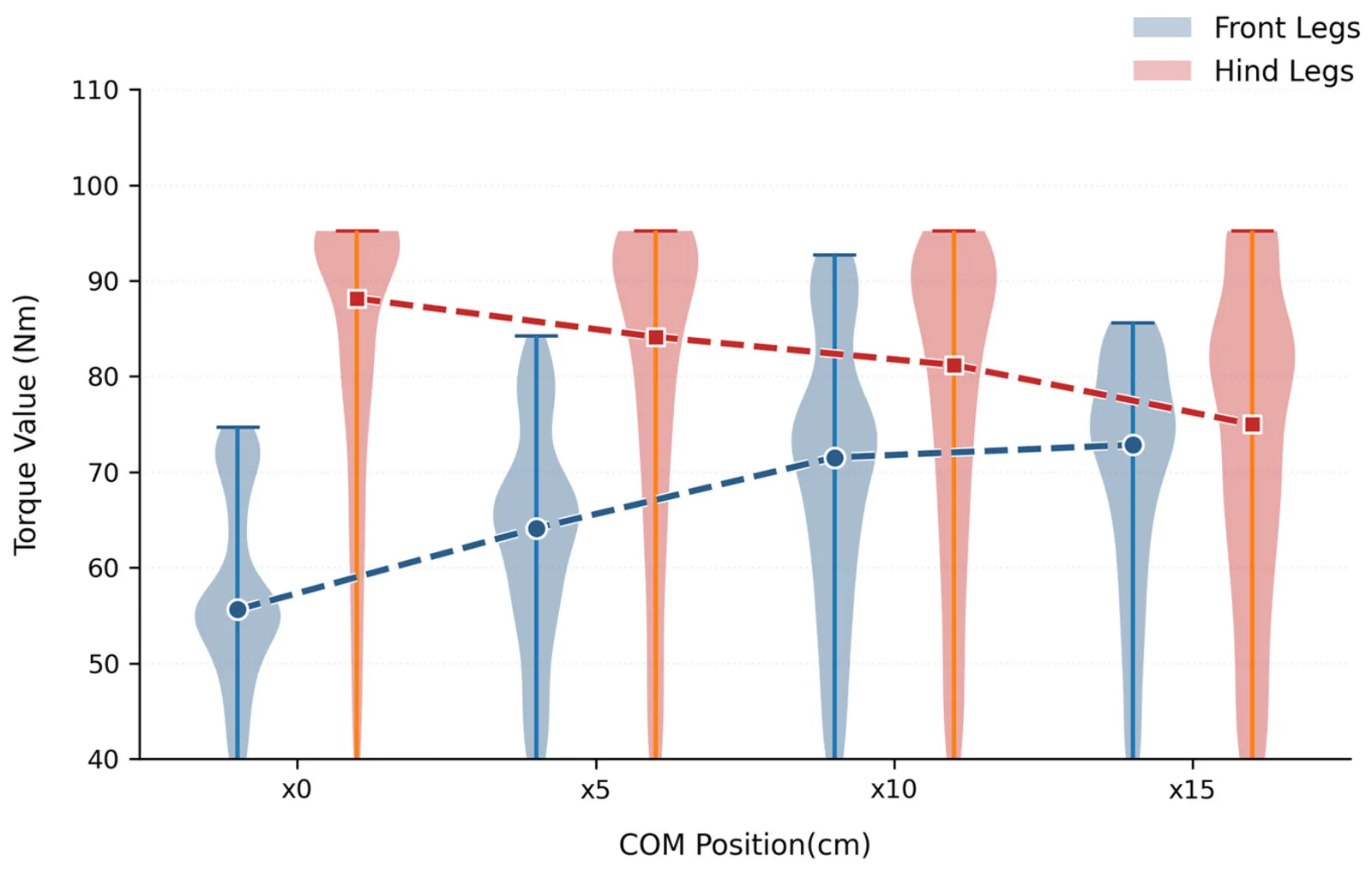
Distribution and statistical characteristics of calf joint torques under different CoM positions. The blue and red dashed lines connect the median values of the forelimb and hindlimb distributions, respectively.

This balancing at the support force level is further transmitted to the joint space. According to the Jacobian transpose mapping, the foot-end ground reaction forces are converted into load torques at the hip and knee (calf) joints. Therefore, as the normal support forces redistribute between the front and rear limbs, a corresponding balancing trend should also emerge in the joint torques. The torque distribution results in Figure 6 are highly consistent with this theoretical expectation: under the ×0 condition, the rear limb calf joint torque is markedly higher than that of the front limb, indicating that the rear limb motor operates in a higher load state. As the CoM shifts progressively forward, the front limb joint torque increases gradually while the rear limb joint torque decreases continuously, and the median torque values of the front and rear limbs converge. Quantitatively, at 5 m/s, the absolute difference between the fore- and hindlimb median support forces decreased from 115.96 N under ×0 to 15.57 N under ×15, while the corresponding absolute difference between the median joint torques decreased from 32.53 to 2.15 N·m, representing reductions of 86.6% and 93.4%, respectively. These results demonstrate that the forward CoM shift progressively redistributes the load from a hindlimb-dominated state toward more balanced fore–hindlimb load sharing, thereby alleviating the load concentration on the hindlimbs.

The above results indicate that the longitudinal forward shift of the CoM does not simply alter the robot posture, but directly reconstructs the underlying dynamic load structure during straight-line locomotion through the transmission chain of “support force redistribution → joint torque redistribution.” For high-mobility straight-line locomotion, this reconstruction is of considerable significance: when the rear limb bears excessive support force and driving torque, the rear limb motors are more prone to reaching the torque saturation boundary. After the CoM shifts forward, the front limb can assume more load, the rear limb torque demand is reduced, and the torque margin of the system is released.

### 3.3. Energy Consumption and Extreme Angular Impulse

**Figure 7:**
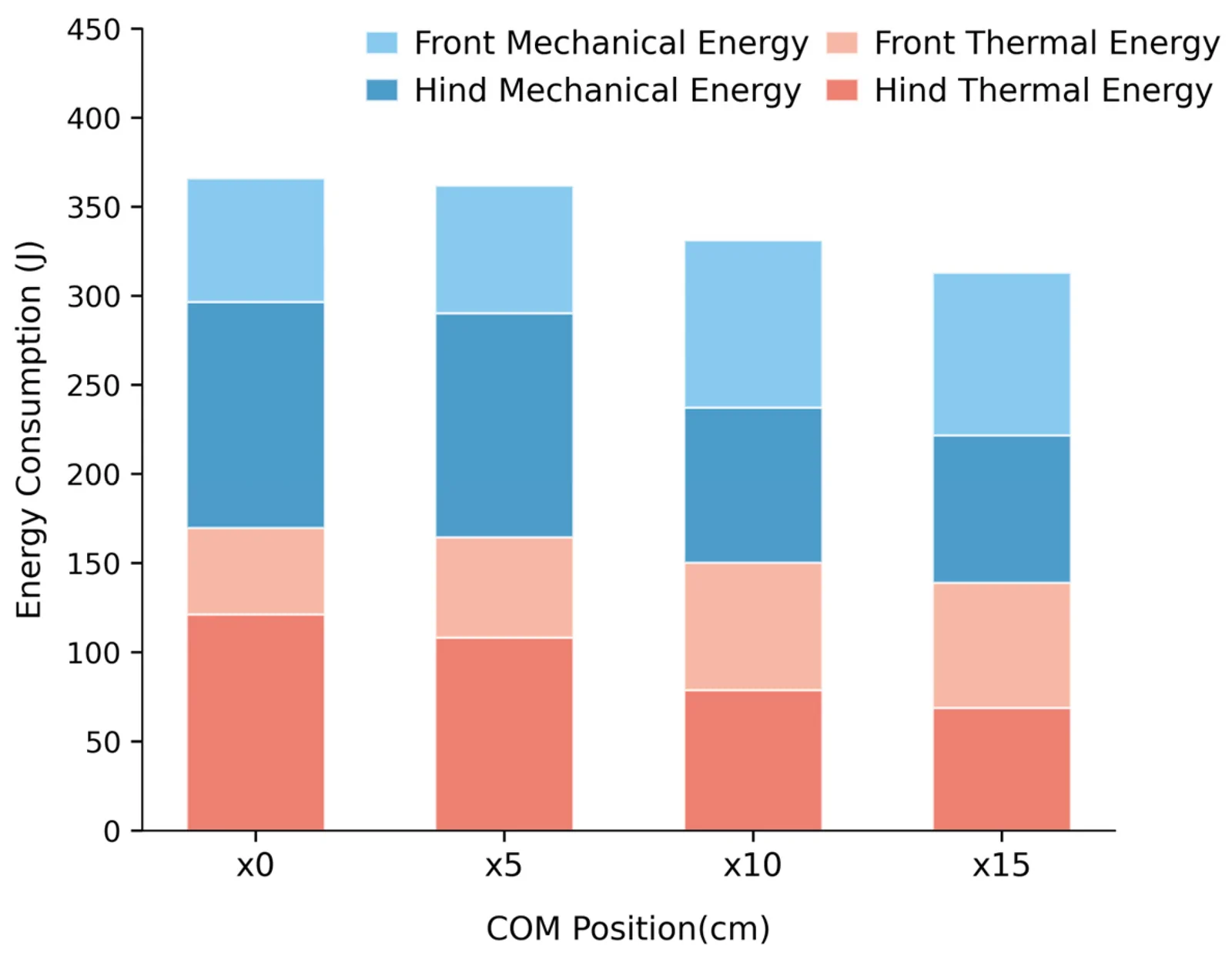
System thermal energy loss and mechanical work under different CoM positions.

The energy consumption results further demonstrate the effect of the fore–hindlimb joint load redistribution. As shown in Figure 7, the total energy consumption is decomposed into two components: motor thermal loss and mechanical work. 

The motor thermal loss is calculated based on the copper loss model [43]. The copper loss of the motor can be expressed as

(20) $$Q_{cu}=I^{2}Rt$$

where I is the motor current and R is the motor resistance, and t is the duration. According to the relationship between the motor current and joint torque,

(21) $$I=\frac{\tau }{K_{t}}$$

the copper loss can be further expressed as

(22) $$Q_{cu}=\frac{R}{{K_{t}}^{2}}\tau^{2}t$$

Therefore, the averaged thermal energy loss over multiple gait cycles is calculated as

(23) $$\frac{1}{N_{c}}\sum_{c=1}^{N_{c}}\sum_{i=1}^{N}\frac{R}{{K_{t}}^{2}}\tau_{c,i}^{2}\Delta t$$

where $N_{c}$ denotes the number of statistical gait cycles, N denotes the number of sampling points within one gait cycle, and Δt = 0.005 s is the sampling interval.

For the motor used in this study, the pole pairs, permanent magnet flux linkage, and gear reduction ratio are p = 21, $\psi_{f}$ = 0.0044 Wb, and i = 8.5, respectively. Ignoring transmission losses, the equivalent output-side torque constant is calculated as

(24) $$K_{t}=\frac{3}{2}p\psi_{f}i=1.1781\;N\cdot m/A$$

With the motor resistance R=0.1Ω, the thermal loss coefficient is

(25) $$k=\frac{R}{{K_{t}}^{2}}=\frac{0.1}{1.1781^{2}}\approx 0.07205$$

Thus, the thermal loss term can be written as

(26) $$\frac{1}{N_{c}}\sum_{c=1}^{N_{c}}\sum_{i=1}^{N}k\tau_{c,i}^{2}\Delta t$$

The mechanical work is obtained by integrating the product of joint torque and joint angular velocity:

(27) $$\frac{1}{N_{c}}\sum_{c=1}^{N_{c}}\sum_{i=1}^{N}\left|\tau_{c,i}\omega_{c,i}\right|\Delta t$$

where $N_{c}$ is the number of statistical cycles, N is the number of sampling points within a single gait cycle, and Δt=0.005 s is the sampling time interval. It can be observed from the figure that as the CoM shifts forward from ×0 to ×15, the total energy consumption exhibits an overall decreasing trend, with both the thermal energy loss and the mechanical work of the rear limb decreasing markedly, indicating that the load concentration on the rear limb is effectively alleviated. Meanwhile, the energy share of the front limb increases, suggesting that part of the dynamic load is transferred from the rear limb to the front limb. Therefore, the forward CoM shift not only reduces the energy burden on the rear limb, but also achieves a more balanced energy distribution between the front and rear limbs, thereby reducing the modeled actuation-related energy consumption under the tested locomotion condition.

**Figure 8:**
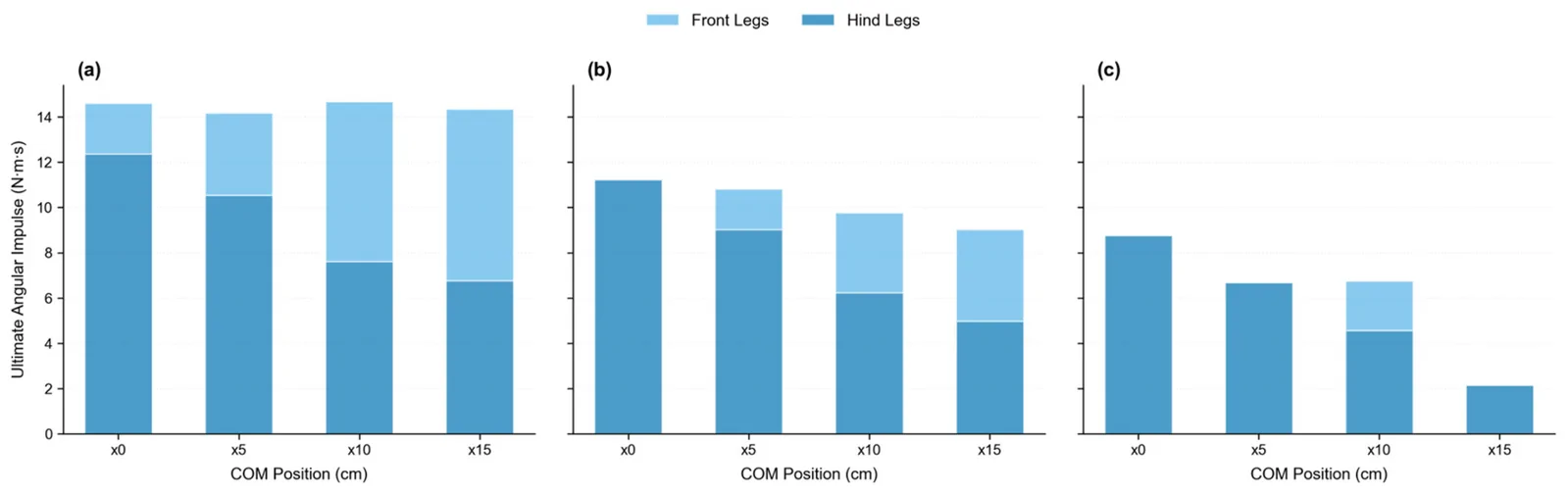
Distribution of extreme angular impulse between the forelimbs and hindlimbs under different longitudinal CoM positions at high-load torque thresholds of (**a**) 70%, (**b**) 80%, and (**c**) 90% of the motor peak torque.

Figure 8 presents the extreme angular impulse results, which characterize the cumulative impact demand on the joints within the high-load torque region. It is calculated as

(28) $$\frac{1}{N_{c}}\sum_{c=1}^{N_{c}}\sum_{i\in \Omega_{lim}}\left|\tau_{c,i}\right|\Delta t$$

where $\Omega_{lim}$ denotes the high-load torque region satisfying $|\tau |\;>\tau_{th}$. The peak torque of the motor used in this study is 95.2 N·m. Accordingly, 70%, 80%, and 90% of the peak torque, corresponding to 66.64, 76.16, and 85.68 N·m, respectively, were selected as the high-load torque thresholds to examine the consistency of the above conclusions under different threshold settings. As shown in Figure 8, under all three thresholds, the extreme angular impulse of the ×0 configuration is dominated by the rear limbs. As the CoM shifts forward, the rear limb extreme angular impulse exhibits an overall decreasing trend, indicating that the forward CoM shift consistently alleviates the high-load state of the rear limbs under different thresholds. The front-limb extreme angular impulse generally increases under the 70% and 80% thresholds, whereas under the 90% threshold it appears only in some CoM configurations. This indicates that the cumulative exposure of the front limbs to torque levels close to the motor peak torque is relatively limited, making the corresponding results more sensitive to the threshold selection. The total extreme angular impulse generally decreases with the forward CoM shift under the 80% and 90% thresholds, whereas under the 70% threshold the results remain similar among the different CoM configurations and do not exhibit a clear monotonic trend. This is because the lower threshold includes a broader and longer-duration range of moderate-to-high torque, such that the reduction in rear limb extreme angular impulse is largely offset by the increase in the front-limb contribution. Overall, the results obtained under different thresholds show that shifting the CoM forward reduces the rear limb extreme angular impulse and redistributes the high-load demand between the front and rear limbs, further confirming its effectiveness in alleviating rear limb high-load conditions.

Overall, the straight-line locomotion results at 5 m/s show that shifting the CoM forward redistributes part of the load from the hindlimbs to the forelimbs, reduces hindlimb joint loading, and promotes a more balanced fore–hindlimb load distribution. The energy consumption and extreme angular impulse results further indicate a reduction in the hindlimb load burden, although the variation in the total extreme angular impulse depends partly on the selected high-load torque threshold. To examine whether the load-redistribution trend remains consistent at a higher speed and across independent training runs, as well as whether the forward CoM shift adversely affects turning response or rough-terrain locomotion stability, additional evaluations are presented in Section 3.4.

### 3.4. Additional Validation and Robustness Analysis

To extend the validation scope of the original high-speed straight-line locomotion results at 5 m/s, and to investigate the effects of longitudinal CoM shifts under different locomotion speeds, motion tasks, terrain conditions, and reinforcement learning training variability, this section further presents evaluations of high-speed straight-line locomotion at 8 m/s, turning performance, randomly generated rough-terrain locomotion, and independent training runs with multiple random seeds.

#### 3.4.1. High-Speed Straight-Line Locomotion at 8 m/s

To examine whether the effect of the longitudinal CoM position on load distribution remains consistent at a higher locomotion speed, additional straight-line locomotion tests were conducted under a constant forward-velocity command of 8 m/s. The fore–hindlimb normal support force and joint torque distributions were compared across the different CoM configurations.

**Figure 9:**
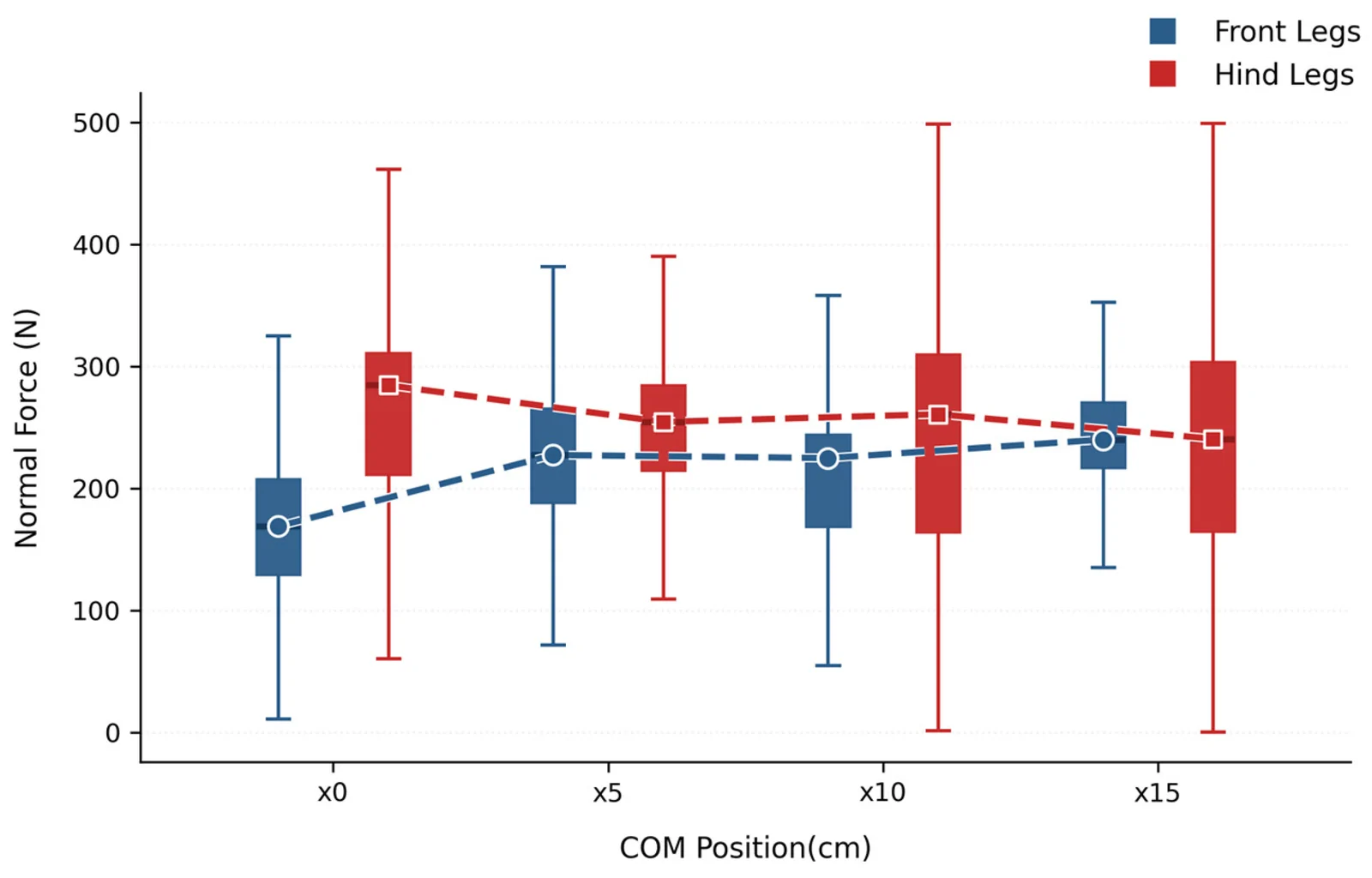
Fore–hindlimb normal support force distributions at 8 m/s under different longitudinal CoM positions. The blue and red dashed lines connect the median values of the forelimb and hindlimb distributions, respectively.

**Figure 10:**
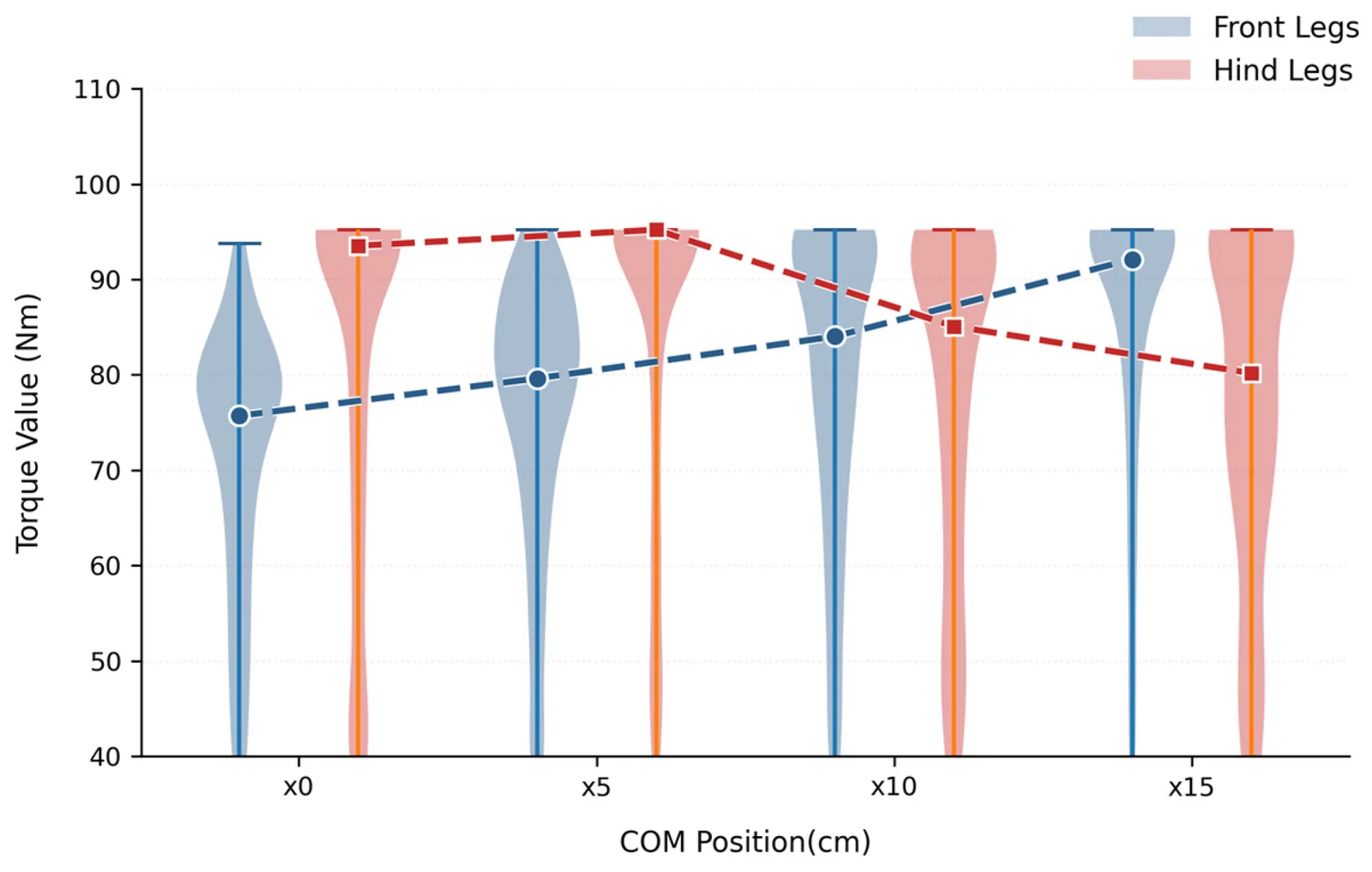
Fore–hindlimb joint torque distributions at 8 m/s under different longitudinal CoM positions. The blue and red dashed lines connect the median values of the forelimb and hindlimb distributions, respectively.

As shown in Figure 9 and Figure 10, the results obtained at 8 m/s exhibit an overall trend consistent with that observed at 5 m/s. Under the ×0 configuration, where the CoM is located at the geometric center of the body, both the normal support force and joint torque of the hindlimbs are higher than those of the forelimbs. As the CoM shifts progressively forward from ×0 to ×15, the forelimb normal support force and joint torque generally increase, whereas the corresponding hindlimb quantities decrease, resulting in a gradual reduction in the fore–hindlimb load difference.

As shown in Figure 10, the fore- and hindlimb joint torques become relatively similar near the ×10 configuration. When the CoM is further shifted forward to ×15, the forelimb joint torque exceeds that of the hindlimbs, indicating that an excessive forward shift causes the load to be redistributed further toward the forelimbs. These results demonstrate that the fore–hindlimb load-redistribution trend induced by a forward CoM shift remains valid at 8 m/s. They also indicate that a more anterior CoM position is not necessarily always advantageous and that the CoM position associated with a more balanced load distribution depends on the specific locomotion condition.

#### 3.4.2. Turning Performance

**Figure 11:**
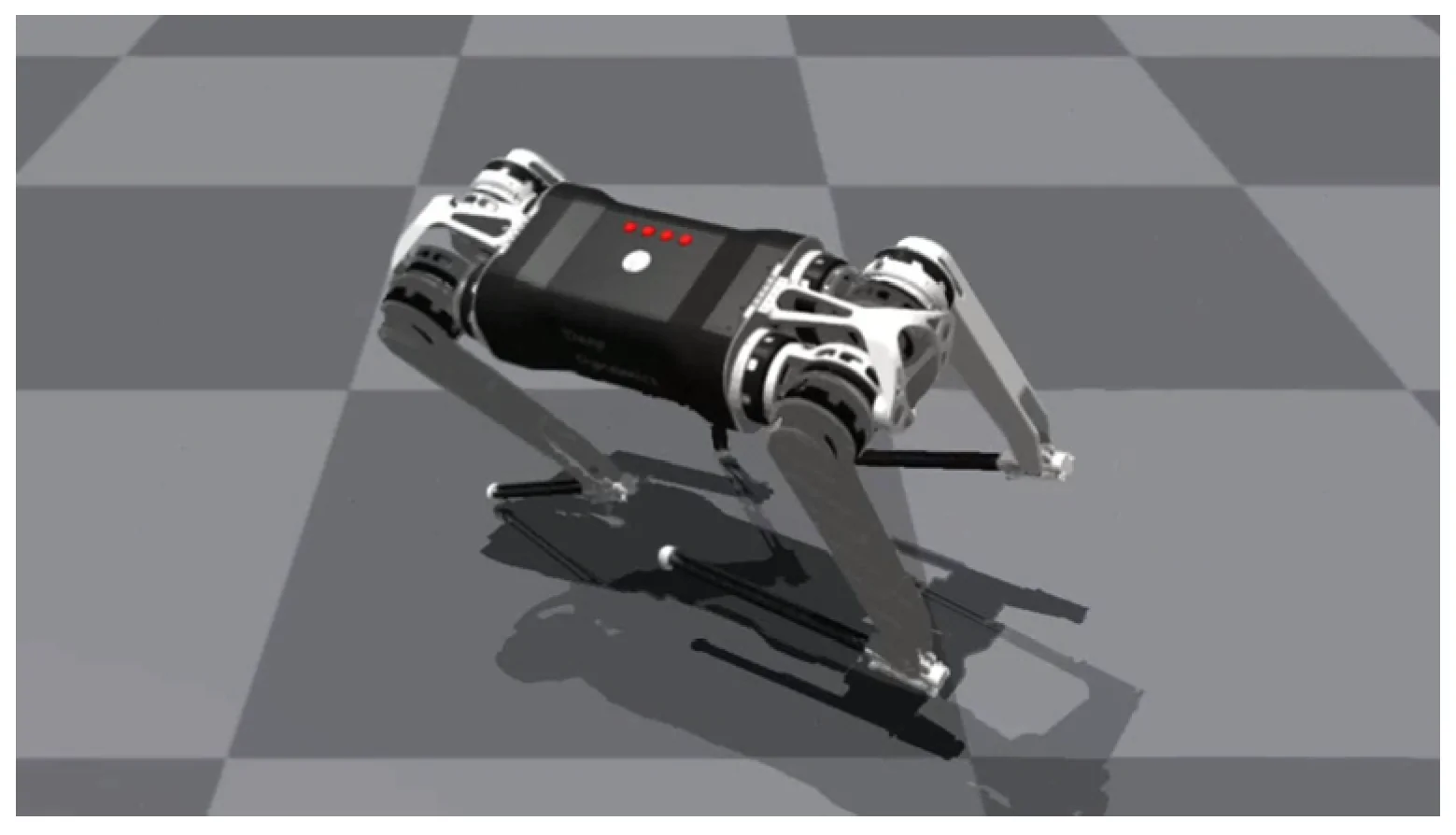
Simulation scene for the high-speed turning evaluation.

**Figure 12:**
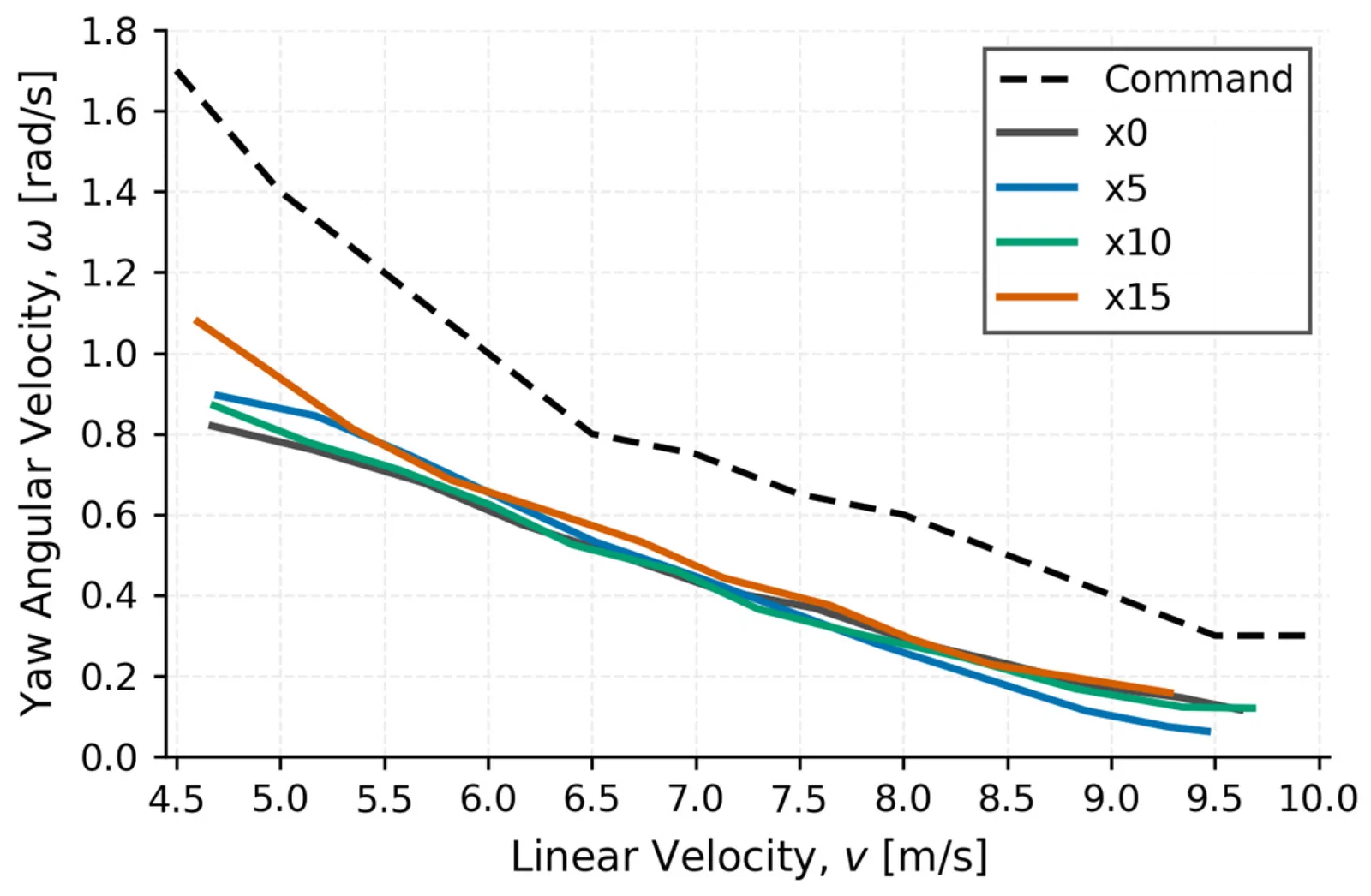
Linear-velocity–yaw-rate response curves under different longitudinal CoM positions.

To evaluate whether the forward longitudinal CoM shift adversely affects high-speed turning performance, the actual yaw-rate responses of the different CoM configurations were measured over a range of commanded forward velocities. The turning test scenario is shown in Figure 11, and the resulting linear-velocity–yaw-rate response curves are presented in Figure 12.

As shown in Figure 12, the actual yaw rate of each CoM configuration generally decreases as the forward velocity increases, reflecting the stronger dynamic constraints on turning response during high-speed locomotion. The linear-velocity–yaw-rate response curves of the different CoM configurations remain close to one another, and no evident contraction of the response range caused by the forward CoM shift is observed in the high-speed regime. Therefore, within the tested ranges of forward-velocity and turning commands, the forward longitudinal CoM shift does not lead to an evident degradation in high-speed turning performance.

#### 3.4.3. Rough-Terrain Stability

**Figure 13:**
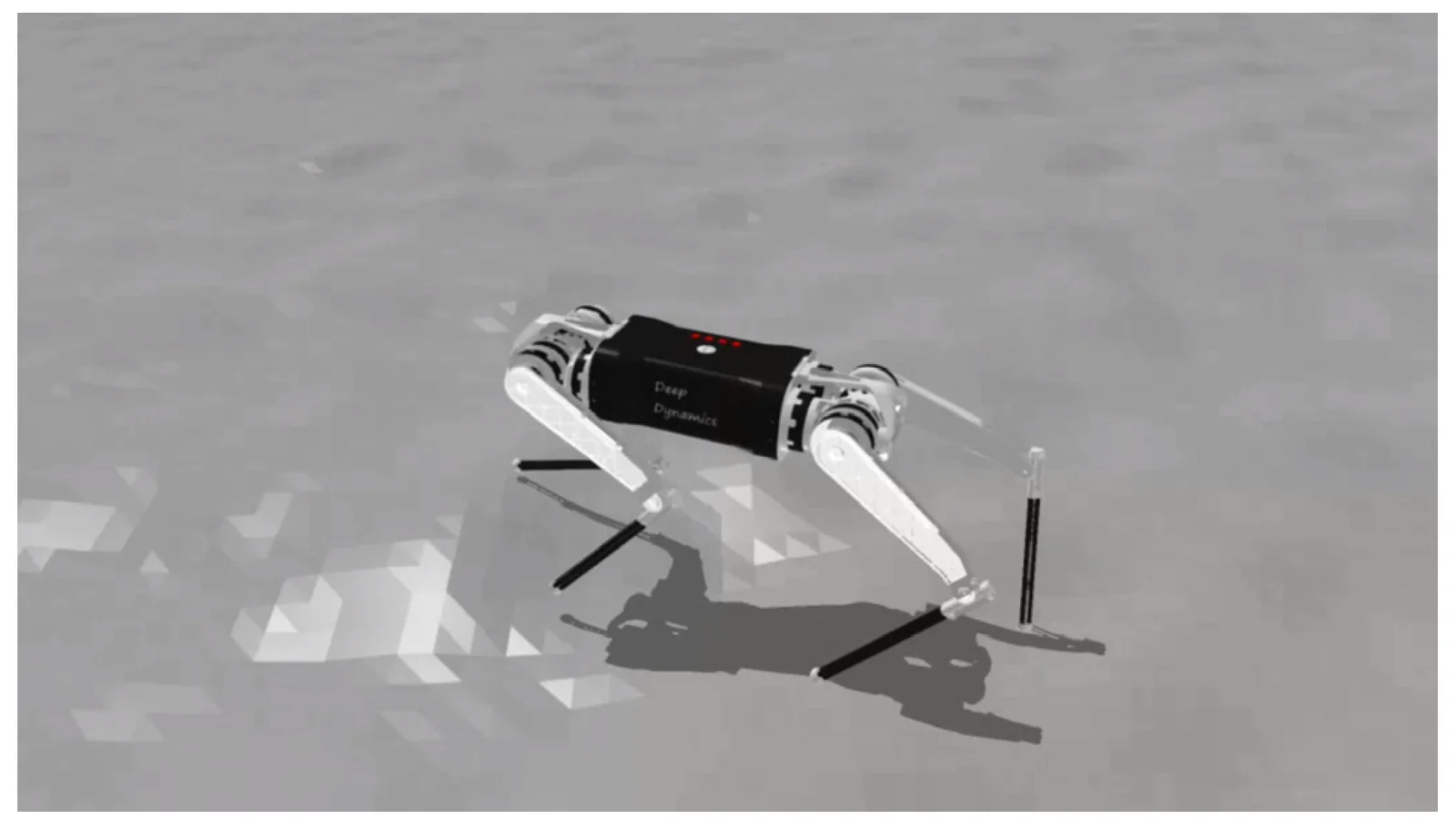
Simulation scenario for the randomly generated rough-terrain evaluation.

To further evaluate the locomotion performance of the different CoM configurations on non-flat terrain, the control policies trained exclusively on flat terrain were directly deployed on a randomly generated rough height field without terrain-specific retraining. The terrain was generated by superimposing smooth random noise and small-amplitude high-frequency perturbations to produce continuous and irregular surface variations. The test scenario is shown in Figure 13.

**Figure 14:**
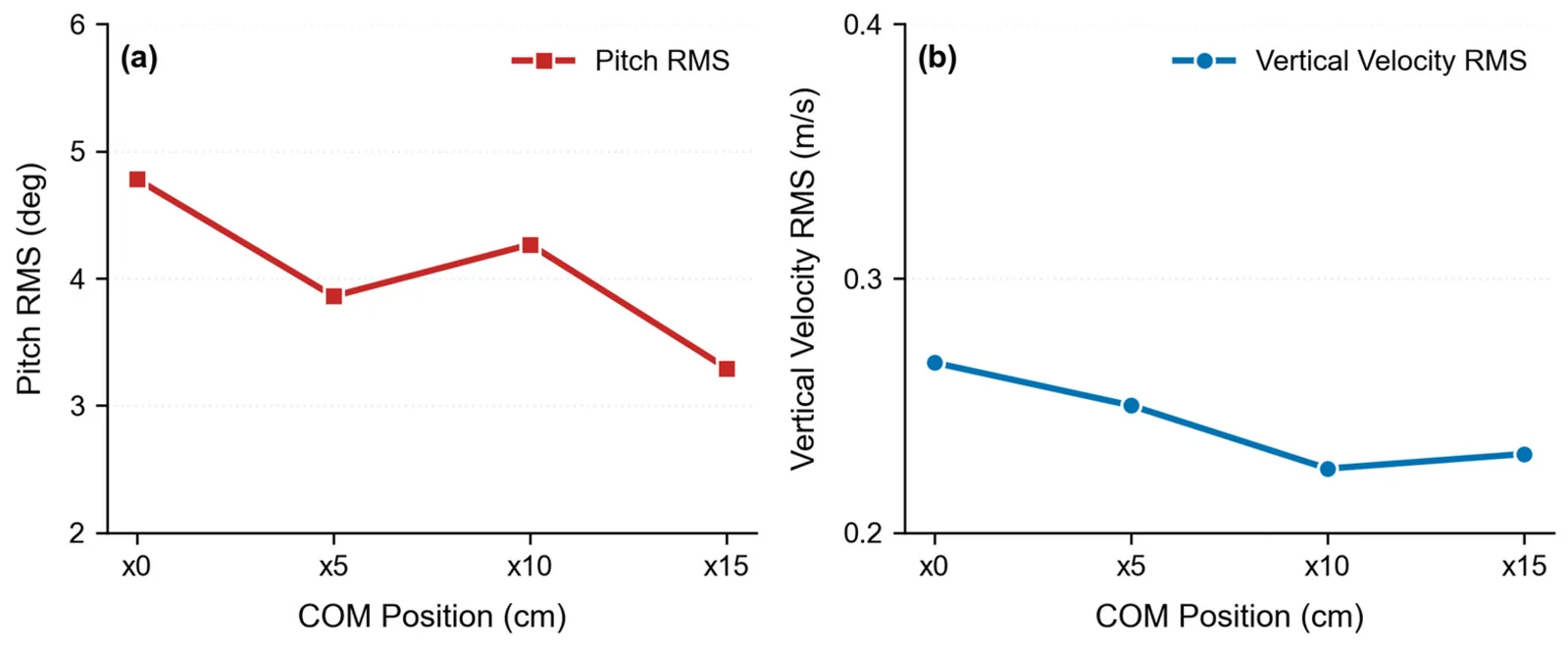
(**a**) Pitch RMS and (**b**) vertical-velocity RMS under different longitudinal CoM positions on randomly generated rough terrain.The pitch RMS and vertical-velocity RMS values remain within similar ranges across the different CoM configurations and do not exhibit an evident increase as the CoM shifts forward. These results indicate that, under the randomly generated rough-terrain conditions considered in this study, the load redistribution induced by the forward longitudinal CoM shift is not accompanied by an evident deterioration in body pitch stability or vertical motion stability.

The root mean square values of the body pitch angle (pitch RMS) and vertical velocity (vertical-velocity RMS) were used to characterize the body pitch fluctuations and vertical motion fluctuations of the robot on the randomly generated rough terrain. The results are presented in Figure 14.

#### 3.4.4. Robustness to Training Randomness

Because each CoM configuration was trained independently, six training runs with random seeds 1–6 were conducted for each of the ×0, ×5, ×10, and ×15 configurations to assess the influence of training variability. All policies were evaluated under the same straight-line locomotion condition. For each run, the median support force and median joint torque during steady-state locomotion were used as representative values; Table 2 reports the mean ± standard deviation of these per-run medians across the six random seeds.

As shown in Table 2, shifting the CoM forward from ×0 to ×15 generally increases the forelimb support force and joint torque while reducing the corresponding hindlimb quantities, thereby progressively narrowing the fore–hindlimb load difference. Despite the variability across random seeds, the cross-seed means of the per-run median support force and joint torque values exhibit the same overall trend as the original single-run results. This indicates that the observed load redistribution remains consistent across different training randomizations and is not an artifact of a single reinforcement learning training run.

## 4. Discussion

Recent studies have improved quadruped locomotion from several complementary perspectives. Jin et al. [44] developed online payload identification and adaptive compensation for unknown payload effects and validated the method on the physical Kirin robot across different payload masses and placements. Dinev et al. [45] jointly optimized robot morphology, payload distribution, actuator parameters, and motion plans, whereas Bjelonic et al. [31] co-designed a parallel-elastic knee mechanism and its controller to reduce torque demand and improve efficiency. Margolis et al. [12], by contrast, focused on reinforcement learning-based high-speed locomotion and sim-to-real transfer on the MIT Mini Cheetah. Table 3 provides a qualitative and quantitative comparison of these studies with the present work.

Taken together, these studies demonstrate the value of adaptive control, morphology and control co-design, compliant actuation, and high-speed policy learning. Their design variables and evaluation protocols differ from those of the present work: the cited studies focus on unknown payload adaptation, joint optimization of multiple design and motion variables, compliant knee actuation, and rapid locomotion control, respectively. In the present study, the robot geometry, total mass, link inertia tensors, joint locations, controller architecture, and training settings are held constant, while only the longitudinal inertial origin of the central body link is shifted. Under the primary 5 m/s evaluation condition, shifting the CoM from ×0 to ×15 reduced the absolute differences between the fore- and hindlimb median support forces and joint torques by 86.6% and 93.4%, respectively. The independent training results obtained with multiple random seeds exhibited the same overall load-redistribution trend, indicating that the observed behavior was robust to reinforcement learning variability. Because the robot scale, actuator capability, locomotion task, environment, and reported metrics differ across studies, the quantitative values in Table 3 should not be interpreted as a direct cross-platform performance ranking. The distinctive contribution of the present work is the controlled isolation and mechanical explanation of longitudinal-CoM-induced load redistribution, which complements existing control- and co-design-oriented approaches.

Despite the systematic investigation of how the longitudinal CoM position affects the dynamic performance of quadruped robots during high-speed straight-line locomotion, the present theoretical model and simulation validation remain subject to specific applicability limits. The findings should therefore be interpreted in relation to the robot platform and locomotion conditions considered.

First, the theoretical framework is based primarily on sagittal-plane dynamics and simplified whole-body force and pitch moment equilibrium. It focuses on the basic mechanical relationships among the longitudinal CoM position, fore–hind ground reaction force distribution, and joint loading. Lateral–yaw coupling, compliant foot–ground contact, structural compliance, and foot-impact effects are not explicitly modeled. Accordingly, the framework is intended to explain the mechanism and overall trend by which the longitudinal CoM position influences fore–hind load distribution, rather than to predict absolute performance accurately under complex three-dimensional locomotion conditions.

Second, trot, bound, and gallop exhibit different foot-contact sequences, aerial phases, and body-motion characteristics, whereas the current model does not explicitly describe contact-phase transitions and the associated transient dynamics over a complete gait cycle. The present results therefore reveal the basic pathway through which the longitudinal CoM position influences fore–hind load distribution but do not provide gait-specific quantitative predictions. A quantitative comparison across gaits will require, building on the present analytical approach, phase-dependent dynamic models that incorporate the corresponding contact transitions, aerial phases, and transient body motion, together with dedicated validation for each gait.

Third, the model is formulated parametrically and does not incorporate empirical relationships specific to the Black Panther 2 platform. The CoM height, the CoM positions relative to the fore and hind feet, body geometry, and foot Jacobians are represented by general variables. Consequently, the pathway by which the longitudinal CoM position changes the fore–hind moment arms and thereby affects ground reaction force and joint load distribution is not unique to the current platform and has a degree of cross-platform mechanical relevance. Nevertheless, the simulations are currently validated only on Black Panther 2, and no cross-morphology experiments have quantified the sensitivity of the results to body size, mass distribution, leg structure, or actuation capability. The present findings therefore support a general mechanism by which longitudinal CoM position affects fore–hind load allocation, rather than an identical improvement magnitude or a universal best CoM position across robot platforms. The results should be regarded as mechanically informed trends and morphology-design insights with potential cross-platform relevance; their quantitative effects must still be evaluated against the structural parameters and physical constraints of each robot.

Fourth, the trunk collision-box length is L=0.50 m, and the ×0, ×5, ×10, and ×15 configurations correspond to central-body inertial-origin offsets of 0, 5, 10, and 15 cm, respectively, equivalent to approximately 0, 0.1 *L*, 0.2 *L*, and 0.3 *L*. A 5 cm increment was selected to span relatively small, moderate, and large adjustments of the central-body mass distribution within the investigated range and thereby provide a coarse discrete sensitivity comparison. These configurations are intended to reveal the overall progression of fore–hind support force and joint torque redistribution and to show that the geometric body center does not necessarily coincide with the dynamically balanced load position; they are not intended to identify any sampled configuration as a global optimum. The discrete results also show that further increasing the forward shift does not necessarily continue to improve fore–hind load balance; thus, the CoM position should not be interpreted as being better simply because it is farther forward. The position associated with a more balanced load state also varies with the locomotion condition. The central finding is therefore that a moderate forward CoM shift can regulate and improve fore–hind load distribution, rather than that one fixed offset applies to all conditions. Because intermediate positions between ×5, ×10, and ×15 and offsets beyond 15 cm were not tested separately, finer continuous parameter scans and task-specific multi-objective optimization that jointly consider peak joint torque, energy consumption, locomotion stability, velocity tracking, and actuator constraints remain for future work.

Fifth, the supplementary turning and randomly generated rough-terrain tests assess potential performance trade-offs of a forward CoM shift from the perspectives of directional response and adaptation to complex terrain, but they do not cover the robot’s full maneuvering envelope. Emergency stopping, lateral jumping, aggressive gait transitions, and recovery from strong external disturbances require further validation. Moreover, shifting the longitudinal CoM forward reduces hindlimb support force and joint torque while increasing the load carried by the forelimbs. The support force, joint torque, and extreme angular impulse metrics used here characterize changes in mechanical loading but cannot directly predict foot wear, structural fatigue, or actuator life during long-term physical operation. These issues require durability experiments on a physical robot.

Finally, multiple independent random-seed training runs were conducted for each CoM configuration to reduce the influence of reinforcement learning randomness, but all current validations remain simulation-based. Contact dynamics, structural compliance, actuator dynamics, sensing errors, and model-parameter discrepancies on a physical robot may alter the quantitative results. Future work will combine physical-robot experiments, continuous CoM parameter scans, and systematic evaluations across robot morphologies, gait types, speed conditions, and highly dynamic tasks to further establish the cross-platform applicability and practical engineering boundaries of longitudinal CoM adjustment.

## 5. Conclusions

This study investigates the influence of longitudinal center of mass (CoM) position on fore–hindlimb load distribution during high-speed straight-line locomotion of quadruped robots. By integrating whole-body force and pitch moment equilibrium, sagittal-plane kinematic analysis, and Jacobian-based foot-force-to-joint-torque mapping, a mechanical relationship is established among the longitudinal CoM position, fore–hindlimb support force distribution, and joint loading. The theoretical analysis shows that, even when the CoM is located at the geometric center of the body, the horizontal ground reaction forces generated during high-speed forward locomotion may produce an additional pitching moment, causing the hindlimbs to sustain greater vertical support forces and joint torques. A moderate forward shift of the CoM alters the moment arm relationships between the CoM and the fore and hind foot-contact locations, thereby redistributing the loads between the fore and hindlimbs.

Simulation results obtained using the Black Panther 2 quadruped robot validate the proposed mechanism. In the primary evaluation at 5 m/s, shifting the CoM from ×0 to ×15 reduced the absolute differences between the fore- and hindlimb median support forces and joint torques by approximately 86.6% and 93.4%, respectively, indicating a transition from hindlimb-dominated loading toward cooperative load sharing between the fore and hindlimbs. The energy results further show that the hindlimb thermal loss and mechanical work decrease as the CoM shifts forward. Under different high-load torque thresholds, the hindlimb extreme angular impulse also exhibits an overall decreasing trend. The 8 m/s evaluation shows a consistent load-redistribution pattern; however, when the CoM is further shifted to ×15, the forelimb joint torque exceeds that of the hindlimbs, indicating that a more anterior CoM position is not necessarily always advantageous.

Independent training runs with multiple random seeds further demonstrate that the observed load-redistribution trend is not attributable to the randomness of a single reinforcement learning training run. Under the tested turning and randomly generated rough-terrain conditions, the forward CoM shift does not cause an evident deterioration in turning response or locomotion stability. Overall, the longitudinal CoM position is not merely a geometric design parameter, but an important morphological factor affecting fore–hindlimb dynamic load distribution in high-speed quadruped locomotion. A moderate forward CoM shift can alleviate hindlimb load concentration and promote a more balanced fore–hindlimb load distribution, providing a theoretical basis for mass-distribution design and the coordinated optimization of morphology and control in high-speed quadruped robots.

## Figures and Tables

**Table 1:** Reward functions and their corresponding weights.

Reward Term	Reward Function *r*_i_	Weight *w*_i_
Linear-velocity tracking	exp{−‖*v_xy_^cmd^* − *v_xy_*‖^2^}	1.0
Yaw-rate tracking	exp{−(*ω_z_^cmd^* − *ω_z_*)^2^}	0.5
Vertical velocity	*v_z_* ^2^	−2.0
Joint acceleration	Σ*j* [($\dot{q_{j,t}}$ − $\overset{˙}{q_{j,t-1}}$)/Δt]^2^	−2.5 × 10^−7^
Base height	(*h* − h*)^2^	−5.0
Base orientation	‖*g_x__y_*‖^2^	−1.0
Joint-position limits	Σ*j* [max(*q_j_^min^* − *q_j_*, 0) + max(*q_j_* − *q_j_^max^*, 0)]	−10.0
Collision	Σ*k_∈{thigh, body}_* 𝟙(‖*F_k_*‖ > 0.1 N)	−1.0
Action smoothness	‖*a_t_* − 2*a*_*t*−1_ + *a*_*t*−2_‖^2^	−0.01
Action rate	‖*a_t_* − *a*_*t*−1_‖^2^	−0.005
Hip-abduction angle	Σ*j_∈hip_ q_j_*^2^	−1.0

Note: h* = 0.58 m.

**Table 2:** Cross-seed statistics of per-run median support forces and joint torques under different CoM configurations.

CoM Position	Forelimb Support Force (N)	Hindlimb Support Force (N)	Forelimb Joint Torque (N·m)	Hindlimb Joint Torque (N·m)
×0	149.87 ± 14.46	256.12 ± 17.15	60.63 ± 7.66	88.55 ± 7.44
×5	168.50 ± 18.65	230.17 ± 19.92	66.56 ± 4.75	82.12 ± 6.00
×10	194.42 ± 17.05	226.28 ± 15.73	70.64 ± 2.71	78.86 ± 5.42
×15	196.08 ± 16.85	200.35 ± 27.12	72.85 ± 2.17	77.30 ± 3.95

Note: For each independent training run, the median support force and median joint torque were calculated from the steady-state evaluation data. Values are reported as the mean ± standard deviation of the six per-run medians.

**Table 3:** Qualitative and quantitative comparison with recent related studies.

Study	Focus and Reported Quantitative Evidence	Relation to the Present Study
Jin et al. [44]	Online identification and adaptive compensation of unknown payload effects; verified on the physical Kirin robot with payload masses of 20–75 kg, together with tests at different torso locations.	Focuses on compensating uncertainty induced by unknown payloads, whereas the present study intentionally varies the nominal longitudinal CoM position to examine fore–hind load redistribution.
Dinev et al. [45]	Bilevel co-design of morphology, payload distribution, actuator parameters, and motion; demonstrated through quadruped trot and jump design tasks and yielding a more energy-efficient Solo design.	Jointly optimizes multiple design and motion variables, whereas the present study holds the remaining morphology and experimental settings constant and isolates the longitudinal CoM position.
Bjelonic et al. [31]	Co-design of a parallel-elastic knee and controller for ANYmal; torque-square efficiency improved by 33%, maximum joint torque decreased by 30%, and flat-terrain operation time increased by 11%.	Modifies knee elasticity to reduce actuator loading, whereas the present study modifies longitudinal mass distribution.
Margolis et al. [12]	RL-based rapid locomotion on the physical MIT Mini Cheetah; 3.9 m/s on flat ground and an average of 3.4 m/s over a 10 m dash on uneven outdoor terrain.	Develops rapid locomotion control and sim-to-real transfer on the MIT Mini Cheetah, whereas the present study evaluates intentional longitudinal CoM offsets.
Our work	Central-body inertial-origin offsets of 0, 0.1 L, 0.2 L, and 0.3 L, evaluated through simulations at 5 and 8 m/s. In the primary evaluation at 5 m/s, shifting the CoM from ×0 to ×15 reduced the absolute median fore–hindlimb differences in support force and joint torque by 86.6% and 93.4%, respectively. Six independent training runs for each CoM configuration further confirmed the consistency of the observed load-redistribution trend.	Varies the longitudinal CoM position while holding the remaining morphology and experimental settings constant, and explains the CoM–ground reaction force–joint load pathway.

Note: Numerical values are reported under different robot platforms, tasks, and metrics and are included to compare validation scopes rather than to rank absolute performance across platforms.

## Data Availability

The data supporting the findings of this study are included in the article. Further inquiries can be directed to the corresponding authors.

## References

[1] Raibert M.H. (1986). Legged Robots That Balance.

[2] Bledt G., Powell M.J., Katz B., Di Carlo J., Wensing P.M., Kim S. (2018). MIT Cheetah 3: Design and Control of a Robust, Dynamic Quadruped Robot. Proceedings of the 2018 IEEE/RSJ International Conference on Intelligent Robots and Systems (IROS), Madrid, Spain, 1–5 October 2018.

[3] Di Carlo J., Wensing P.M., Katz B., Bledt G., Kim S. (2018). Dynamic Locomotion in the MIT Cheetah 3 Through Convex Model-Predictive Control. Proceedings of the 2018 IEEE/RSJ International Conference on Intelligent Robots and Systems (IROS), Madrid, Spain, 1–5 October 2018.

[4] Hwangbo J., Lee J., Dosovitskiy A., Bellicoso C.D., Tsounis V., Koltun V., Hutter M. (2019). Learning Agile and Dynamic Motor Skills for Legged Robots. Sci. Robot..

[5] Hoeller D., Rudin N., Sako D., Hutter M. (2024). ANYmal Parkour: Learning Agile Navigation for Quadrupedal Robots. Sci. Robot..

[6] Makoviychuk V., Wawrzyniak L., Guo Y., Lu M., Storey K., Macklin M., Hoeller D., Rudin N., Allshire A., Handa A. (2021). Isaac Gym: High Performance GPU-Based Physics Simulation for Robot Learning. arXiv.

[7] Rudin N., Hoeller D., Reist P., Hutter M. (2022). Learning to Walk in Minutes Using Massively Parallel Deep Reinforcement Learning. Proceedings of the 5th Conference on Robot Learning, London, UK, 8–11 November 2021.

[8] Nahrendra I.M.A., Yu B., Myung H. (2023). DreamWaQ: Learning Robust Quadrupedal Locomotion with Implicit Terrain Imagination via Deep Reinforcement Learning. Proceedings of the 2023 IEEE International Conference on Robotics and Automation (ICRA), London, UK, 29 May–2 June 2023.

[9] Kumar A., Fu Z., Pathak D., Malik J. RMA: Rapid Motor Adaptation for Legged Robots. Proceedings of the Robotics: Science and Systems XVII.

[10] Lee J., Hwangbo J., Wellhausen L., Koltun V., Hutter M. (2020). Learning Quadrupedal Locomotion over Challenging Terrain. Sci. Robot..

[11] Miki T., Lee J., Hwangbo J., Wellhausen L., Koltun V., Hutter M. (2022). Learning Robust Perceptive Locomotion for Quadrupedal Robots in the Wild. Sci. Robot..

[12] Margolis G.B., Yang G., Paigwar K., Chen T., Agrawal P. (2024). Rapid Locomotion via Reinforcement Learning. Int. J. Robot. Res..

[13] Han L., Zhu Q., Sheng J., Zhang C., Li T., Zhang Y., Zhang H., Liu Y., Zhou C., Zhao R. (2024). Lifelike Agility and Play in Quadrupedal Robots Using Reinforcement Learning and Generative Pre-Trained Models. Nat. Mach. Intell..

[14] Shafiee M., Bellegarda G., Ijspeert A.J. (2024). Viability Leads to the Emergence of Gait Transitions in Learning Agile Quadrupedal Locomotion on Challenging Terrains. Nat. Commun..

[15] Humphreys J., Zhou C. (2025). Learning to Adapt through Bio-Inspired Gait Strategies for Versatile Quadruped Locomotion. Nat. Mach. Intell..

[16] Watanabe T., Kubo A., Tsunoda K., Matsuba T., Akatsuka S., Noda Y., Kioka H., Izawa J., Ishii S., Nakamura Y. (2025). Hierarchical Reinforcement Learning with Central Pattern Generator for Enabling a Quadruped Robot Simulator to Walk on a Variety of Terrains. Sci. Rep..

[17] Choi S., Ji G., Park J., Kim H., Mun J., Lee J.H., Hwangbo J. (2023). Learning Quadrupedal Locomotion on Deformable Terrain. Sci. Robot..

[18] Kim H., Oh H., Park J., Kim Y., Youm D., Jung M., Lee M., Hwangbo J. (2025). High-Speed Control and Navigation for Quadrupedal Robots on Complex and Discrete Terrain. Sci. Robot..

[19] Jin Y., Liu X., Shao Y., Wang H., Yang W. (2022). High-Speed Quadrupedal Locomotion by Imitation–Relaxation Reinforcement Learning. Nat. Mach. Intell..

[20] Hutter M., Gehring C., Jud D., Lauber A., Bellicoso C.D., Tsounis V., Hwangbo J., Bodie K., Fankhauser P., Bloesch M. (2016). ANYmal—A Highly Mobile and Dynamic Quadrupedal Robot. Proceedings of the 2016 IEEE/RSJ International Conference on Intelligent Robots and Systems (IROS), Daejeon, Republic of Korea, 9–14 October 2016.

[21] Katz B., Di Carlo J., Kim S. (2019). Mini Cheetah: A Platform for Pushing the Limits of Dynamic Quadruped Control. Proceedings of the 2019 International Conference on Robotics and Automation (ICRA), Montreal, QC, Canada, 20–24 May 2019.

[22] Fan Y., Pei Z., Wang C., Li M., Tang Z., Liu Q. (2024). A Review of Quadruped Robots: Structure, Control, and Autonomous Motion. Adv. Intell. Syst..

[23] Hobbs S.J., Richards J., Clayton H.M. (2014). The Effect of Centre of Mass Location on Sagittal Plane Moments around the Centre of Mass in Trotting Horses. J. Biomech..

[24] Hobbs S.J., Clayton H.M. (2022). The Olympic Motto through the Lens of Equestrian Sports. Anim. Front..

[25] Fish F.E., Sheehan M.J., Adams D.S., Tennett K.A., Gough W.T. (2021). A 60:40 Split: Differential Mass Support in Dogs. Anat. Rec..

[26] Lee D.V., Stakebake E.F., Walter R.M., Carrier D.R. (2004). Effects of Mass Distribution on the Mechanics of Level Trotting in Dogs. J. Exp. Biol..

[27] Jayes A.S., Alexander R.M. (1978). Mechanics of Locomotion of Dogs (*Canis familiaris*) and Sheep (*Ovis aries*). J. Zool..

[28] Bishop K.L., Pai A.K., Schmitt D. (2008). Whole Body Mechanics of Stealthy Walking in Cats. PLoS ONE.

[29] Manter J.T. (1938). The Dynamics of Quadrupedal Walking. J. Exp. Biol..

[30] Belmonte-Baeza Á., Lee J., Valsecchi G., Hutter M. (2022). Meta Reinforcement Learning for Optimal Design of Legged Robots. IEEE Robot. Autom. Lett..

[31] Bjelonic F., Lee J., Arm P., Sako D., Tateo D., Peters J., Hutter M. (2023). Learning-Based Design and Control for Quadrupedal Robots with Parallel-Elastic Actuators. IEEE Robot. Autom. Lett..

[32] Chen C., Xiang P., Zhang J., Xiong R., Wang Y., Lu H. (2024). Deep Reinforcement Learning Based Co-Optimization of Morphology and Gait for Small-Scale Legged Robot. IEEE/ASME Trans. Mechatron..

[33] Drăgoi M.-V., Nisipeanu I., Puiu R.-A., Tache F.-G., Spiridon-Mocioacă T.-M., Hank A., Cristoiu C. (2025). An Inclusive Offline Learning Platform Integrating Gesture Recognition and Local AI Models. Biomimetics.

[34] Orin D.E., Goswami A., Lee S.H. (2013). Centroidal Dynamics of a Humanoid Robot. Auton. Robot..

[35] Koolen T., de Boer T., Rebula J., Goswami A., Pratt J. (2012). Capturability-Based Analysis and Control of Legged Locomotion, Part 1: Theory and Application to Three Simple Gait Models. Int. J. Robot. Res..

[36] Caron S., Pham Q.C., Nakamura Y. (2017). ZMP Support Areas for Multicontact Mobility under Frictional Constraints. IEEE Trans. Robot..

[37] Mason M.T. (2001). Mechanics of Robotic Manipulation.

[38] Spong M.W., Hutchinson S., Vidyasagar M. (2006). Robot Modeling and Control.

[39] Murray R.M., Li Z., Sastry S.S. (1994). A Mathematical Introduction to Robotic Manipulation.

[40] Featherstone R. (2008). Rigid Body Dynamics Algorithms.

[41] Schulman J., Wolski F., Dhariwal P., Radford A., Klimov O. (2017). Proximal Policy Optimization Algorithms. arXiv.

[42] Kingma D.P., Welling M. Auto-Encoding Variational Bayes. Proceedings of the 2nd International Conference on Learning Representations (ICLR).

[43] Seok S., Wang A., Chuah M.Y., Otten D., Lang J., Kim S. (2015). Design Principles for Energy-Efficient Legged Locomotion and Implementation on the MIT Cheetah Robot. IEEE/ASME Trans. Mechatron..

[44] Jin B., Ye S., Su J., Luo J. (2022). Unknown Payload Adaptive Control for Quadruped Locomotion With Proprioceptive Linear Legs. IEEE/ASME Trans. Mechatron..

[45] Dinev T., Mastalli C., Ivan V., Tonneau S., Vijayakumar S. (2022). A Versatile Co-Design Approach for Dynamic Legged Robots. Proceedings of the 2022 IEEE/RSJ International Conference on Intelligent Robots and Systems (IROS), Kyoto, Japan, 23–27 October 2022.

